
JOURNAL INFORMATION
==============================
NLM Title Abbreviation: Innov Surg Sci
Journal ID: Innov Surg Sci
NLM Journal ID: 101708165
Journal ID: iss

Innovative Surgical Sciences

EISSN: 2364-7485
Publisher: De Gruyter

ARTICLE INFORMATION
==============================
PMCID: PMC13068492
DOI: 10.1515/iss-2026-2003
Article ID: iss-2026-2003
Article version: 1
Subjects: Article

3. Paediatric Surgery

Electronic publication date: 2026 Apr 10
Publication date: 2026 Apr
Volume: 11
Issue: Suppl1
First page: 270
Last page: 329
Copyright: © 2026 by Walter de Gruyter Berlin/Boston
Copyright year: 2026
Copyright holder: De Gruyter
License: This work is licensed under the Creative Commons Attribution 4.0 International License.
License URL: https://creativecommons.org/licenses/by/4.0/

Page count: 60

==============================

DE GRUYTER BRILL

**Burning Issues - The Reality of Pediatric Burn Care in Germany**
ID: E-DCK-313

**Kinder- und Jugendchirurgie / Paediatric and Adolescent Surgery**
**Aktuelle Behandlungsstrategien bei Thermischen Verletzungen / Latest Approaches to Thermal Injuries**

Sabine Drossard[1], Andrea Schmedding[2]

[1]Universitätsklinikum Würzburg, Klinik und Poliklinik für Allgemein-, Viszeral-, Transplantations-, Gefäß- und Kinderchirurgie, Würzburg, Deutschland, [2]Städtisches Klinikum Braunschweig, Abteilung für Kinderchirurgie und Kinderurologie, Braunschweig, Deutschland

**Background:**
Each year, more than 7,000 children and adolescents aged 0–17 years are hospitalized in Germany due to thermal injuries. Treatment is provided not only in the 19 designated centers for severely burned children but also in specialized pediatric units as well as in non-certified hospitals, involving disciplines such as pediatric surgery and plastic surgery. Burn-injured children place high demands on acute care and surgical management. While there are no standardized treatment algorithms, an S2k guideline has been available since 2024. Although many hospitals contribute their data to the Burn Registry of the German Society for Burn Medicine (*Deutsche Gesellschaft für Verbrennungsmedizin, DGV*), comprehensive information on the actual care reality has so far been lacking. This study provides an overview of the incidence and current treatment structures for thermally injured children and adolescents in Germany, based on an analysis of data from the past five years.

**Material and methods:**
We conducted a retrospective analysis of data from the German Institute for the Hospital Remuneration System (*Institut für das Entgeltsystem im Krankenhaus, InEK*) for the years 2019–2024. These data comprise the cumulative annual statistics of patients insured under the statutory health insurance (*Gesetzliche Krankenversicherung, GKV*). All thermal injuries are coded both by localization and by total burn surface area (ICD code: T31). We included all inpatient cases under 18 years of age with burns or scalds, identified by ICD code T31. Each annual dataset provided information on age distribution, mean length of hospital stay, as well as the main diagnoses (ICD), secondary diagnoses (ICD), procedures (OPS), and DRGs of the cohort. *InEK* does not provide values smaller than four per year; therefore, very small case numbers are not differentiated in this analysis due to the nature of the dataset.

**Results:**
Between 2019 and 2024, an average of 7,530 children and adolescents with thermal injuries were hospitalized each year in Germany, corresponding to a total of 45,179 cases. While the incidence declined steadily from 8,322 patients in 2019 to 6,949 patients in 2022, a gradual increase has been observed since then, reaching 7,317 patients in 2024. Overall, 57% of the affected patients were male. With regard to age distribution, 13.8% of the children were younger than one year, 45.7% were between one and two years old, and 40.5% were three years or older.
In most patients, the burn injury involved only a small proportion of the body surface. Specifically, 89.8% of cases affected less than 10% of the total body surface area (TBSA). Moderate burns, with an extent of

Open Access. © 2026 The Author(s), published by De Gruyter. This work is licensed under the Creative Commons Attribution 4.0 International License.

10–19% TBSA, were observed in 9.1% of cases, while larger injuries of 20–29% TBSA occurred in 1.5%. Extensive burns were rare: only 0.5% of patients presented with burns covering 30–39% TBSA, and just 0.1% had injuries affecting 40–49% TBSA.
In a total of 308 cases (0.7%), an injury caused by electric current was documented. Neglect and abuse were coded as diagnoses in 35 and 59 cases, respectively (0,21%). An evaluation for child welfare endangerment was documented as a procedure in 0.71% of treatment cases.
Intensive care treatment was required in 12% of cases. A central venous catheter was placed in 2% of patients, and blood transfusions were administered in 1%. Overall mortality was 0.08%, but it was markedly higher among adolescents (0.45%).
The most frequently performed interventions included surgical debridement and temporary soft tissue coverage using hydrolytic membranes, the use of which increased from 61% of all cases in 2019 to 74% in 2024. Split-thickness skin grafts were performed in 11%, vacuum-assisted closure therapy in 2%. Escharotomies were required in 106 cases (0.23%). Enzymatic debridement was coded in 0.58% of cases, showing an increase since 2020 and reaching 51 cases in 2024. Tracheoscopy was performed in 0.3% of patients, and esophagogastroduodenoscopy in 0.1%.
Billing data indicated that 53% of cases were coded under pediatric surgery, 37% under pediatrics, 5% under plastic surgery, 4% under trauma surgery, and 3% under general surgery. Patients treated by plastic surgery were on average considerably older (47.1% >10 years vs. 13.6% in pediatric surgery). In the 16–17-year age group, only 20.7% of cases were billed under pediatric surgery. The most frequently coded DRG was Y03Z (“Other burns with other interventions”), with a mean of 3,686 cases per year, corresponding to an average of 2,834 case-mix points annually.

**Conclusion:**
This analysis provides, for the first time, a nationwide overview of the surgical care of burn-injured children and adolescents in Germany and enables an evidence-based discussion on the role of different care structures and potential areas for optimization. Thermal injuries remain a relevant clinical condition in childhood and adolescence in Germany, with no sustainable decline in incidence detectable. In early childhood, treatment is predominantly provided in pediatric surgery and pediatrics departments, whereas adolescents are primarily treated within adult surgical services. However, they require special attention in burn medicine due to their considerably higher mortality. Overall, the analysis highlights the heterogeneity of the disciplines involved and underscores the need for standardized treatment protocols and strengthened interdisciplinary collaboration.

DE GRUYTER BRILL

**Application Options for SpinCare© in Children – An Alternative to Challenging Dressing Procedures**
ID: E-DCK-495

**Kinder- und Jugendchirurgie / Paediatric and Adolescent Surgery**
**Aktuelle Behandlungsstrategien bei Thermischen Verletzungen / Latest Approaches to Thermal Injuries**

Aileen Flach[1], Marcel Noatnick[1], Leila Harhaus-Wähner[2]

[1]Sana Klinikum Lichtenberg, Kinderbrandverletztenzentrum, Kinderchirurgie, Berlin, Deutschland, [2]BG Klinikum Unfallkrankenhaus Berlin, Klinik für Hand-, Replantations- und Mikrochirurgie, Klinik für Schwerbrandverletzte und Plastische Chirurgie, Berlin, Deutschland

**Background:**
A selection of patients treated in-house with SpinCare will be presented. Application areas include the face, head (post-transplantation), genitals, and buttocks. Using a case series of more than 100 patients, the possible applications as well as the advantages and disadvantages of SpinCare use in childhood will be discussed, including the perspective of the nursing staff.

**Material and methods:**
**Retrospective Evaluation of SpinCare Application in Pediatric Patients**
SpinCare is a "matrix of nanofibers produced by electrospinning technology that mimics the biological structure of the human extracellular matrix," offered by Nanomedics.
This paper presents an internal retrospective evaluation of SpinCare application at "difficult" anatomical sites such as the head, face, genitals, and buttocks. These locations are defined as difficult due to the particular challenges they pose for dressing application, hygiene, and patient or parental compliance.
In the anogenital area, dressing application is complicated by the natural excretions of the patient, which can contaminate the otherwise protective dressings and potentially lead to wound infections.
In the face and head regions, dressings often have poor adhesion due to the need to spare eyes, mouth, ears, and nose, which can cause difficulties with breathing, eating, and compliance in children.
Since October 2023, SpinCare has been regularly used at the Children's Burn Center of Sana Klinikum Lichtenberg - in cooperation with the Burn Center BG Clinic Berlin - for children with appropriate indications, resulting in a patient cohort of more than 100 children.
This study aims to describe the application of SpinCare according to internal standards and to outline the advantages and disadvantages of SpinCare compared to conventional dressings using alloplastic materials in second-degree superficial to deep partial-thickness burns (Grade IIa-b).
Furthermore, the perspectives of the nursing staff will be analyzed to assess the advantages and disadvantages of SpinCare in terms of nursing care and parental compliance.

**Results:**
For second-degree burn wounds, SpinCare© proved to be a practical and significantly better-tolerated alternative to traditional dressings. This was especially evident in burns located on the head and face, where SpinCare© led to reduced pain and itching, while also supporting favorable cosmetic outcomes.

**Conclusion:**
Very young children (particularly under 18 months of age) benefit greatly from SpinCare© treatment, as it supports a child-friendly daily routine—including activities such as breastfeeding and bottle feeding. Our evaluation demonstrates that SpinCare© is an effective and well-tolerated alternative for treating burns in anatomically sensitive or hard-to-dress areas.

DE GRUYTER BRILL

### Microbial DNA in Residual Bile Fluid of Biliary Atresia: Composition and Clinical Implications
ID: E-DCK-124

**Kinder- und Jugendchirurgie / Paediatric and Adolescent Surgery**
**Kinderchirurgische Forschung / Paediatric Surgery Research**

Maximilian Holweg[1], Marie Uecker[2], Andreas Schmidt[1], Joachim Kübler[3], Jörg Fuchs[1], Omid Madadi-Sanjani[2], Christoph Slavetinsky[1]

[1]University Children's Hospital Tübingen, Department of Pediatric Surgery and Pediatric Urology, Tübingen, Deutschland, [2]University Medical Center Hamburg-Eppendorf, Department of Pediatric Surgery, Hamburg, Deutschland, [3]Bremen Hospital Group, Department of Pediatric Surgery, Bremen, Deutschland

### Background:
Biliary atresia (BA) is a rare and life-threatening neonatal cholangiopathy, characterized by progressive fibro-inflammatory obliteration of the bile ducts. Surgical treatment involves Kasai portoenterostomy (KPE), yet despite early intervention, up to 80% of patients require liver transplantation due to disease progression. Its etiology remains poorly understood, with proposed contributions from genetic, infectious, and autoimmune factors. Because this condition occurs only in neonates, increasing attention is being paid to how early immune dysregulation and initial intestinal microbial colonization contribute to liver inflammation and fibrogenesis. Understanding this interaction may open new avenues for targeted therapies in BA. Since the disease is localized to the biliary tract, the aim of this study was (i) to determine microbial DNA and its composition in bile fluid of the gall bladder remnant of BA, and (ii) to explore possible implications for disease progression.

### Material and methods:
Bile was collected intraoperatively, as most BA patients retain a gallbladder remnant with residual fluid. BA patients treated at University Hospital Tübingen and Hannover Medical School (2022–2024) were recruited. Age-matched controls were patients without liver disease (normal controls, NC) and with familial cholestasis (disease controls, DC). Exclusion criteria (all groups) were non-harvestable bile; for controls, diagnosed or suspected BA; and overall age >5 years. The study was approved by local Ethics Committees and complied with the Declaration of Helsinki. Twenty patients were enrolled in this pilot study: 14 BA, 3 NC, and 3 DC. Pediatric bile samples were collected under standardized sterile conditions, followed by nucleic acid extraction and 16S rRNA gene sequencing (V4) to profile microbial communities. Bioinformatics used reproducible nf-core pipelines (ampliseq; differential abundance), including denoising (DADA2), taxonomic classification (SILVA 138.1), diversity analyses (QIIME2; Observed Features, Faith PD, Shannon entropy, Pielou's evenness), and differential abundance testing (DESeq2). Further statistics were performed in GraphPad Prism (Kruskal-Wallis, Mann-Whitney U, unpaired t-test).

### Results:
The diversity and composition of microbial DNA varied substantially across patients. Hierarchical clustering revealed distinct patient subgroups that separated BA from DC and NC across phylum, class, order, family, and genus levels. Alpha diversity was significantly lower in BA than in NC (p=0.03) and DC

(p=0.02). At the phylum level, Proteobacteria were increased in BA versus NC and DC (39.4% vs 8.9% vs 12.9%), whereas Bacteroidota were reduced in BA versus NC (14.9% vs 33.0%) and Fusobacteriota were reduced in BA versus NC and DC (0.6% vs 5.8% vs 4.9%). At the class level, Alphaproteobacteria and Gammaproteobacteria were increased in BA versus NC (4.9% vs 0.3%; 34.5% vs 8.6%), while Bacteroidia and Negativicutes were decreased (14.9% vs 33.0%; 0.6% vs 6.3%). Clostridia were prevalent across all groups (BA 27.1%, NC 25.6%, DC 35.5%). Finally, at the class level, Actinobacteria were more abundant in BA patients achieving clearance of jaundice (COJ) than in those without COJ or requiring liver transplantation (LTx) (mean 11.9% vs 3.8%; p=0.049).

**Conclusion:**

This study shows that microbial DNA is present in residual bile fluid in BA and identifies distinct alterations in microbial DNA composition in BA compared to NC and DC. These shifts could mirror reported intestinal dysbiosis in BA. Higher abundance of Actinobacteria DNA in patients achieving COJ suggests prognostic value of specific microbial signatures. Notably, this is the first report of microbial DNA in BA bile fluid, supporting early postnatal colonization of residual bile ducts by intestinal bacteria before complete biliary obliteration, with potential implications for disease progression. While microbiome changes appear to be present in BA, their direct impact on pathophysiology and outcomes remains uncertain. Future work should integrate microbial, immunological, and metabolic data to clarify links between colonization, bile duct injury, and prognosis.

**Impact of Microscopically Positive Resection Margins (R1) on Recurrence/Survival in Hepatoblastoma**
ID: E-DCK-128

**Kinder- und Jugendchirurgie / Paediatric and Adolescent Surgery**
**Kinderchirurgische Forschung / Paediatric Surgery Research**

Ida Susanna Reinhold[1], Patrick Günther[1], Markus Kessler[1], Philipp Romero[1], Fabian Ruping[1], Juri Fuchs[1]

[1]Department of Pediatric Surgery, University Hospital Heidelberg, Heidelberg, Deutschland

**Background:**
Surgical resection remains the cornerstone of curative treatment in pediatric hepatoblastoma. In patients with large or centrally located tumors, however, achieving microscopically complete (R0) resection may be technically impossible or entail excessive surgical risks. While R0 margins are generally regarded as the gold standard, some studies have suggested that R1 (microscopically positive) margins do not necessarily confer a significant survival disadvantage. This issue is particularly relevant for tumors abutting major vascular structures, where the balance between margin width, surgical risk, and the potential indication for liver transplantation becomes critical. The aim of this meta-analysis was to systematically evaluate the prognostic impact of R1 versus R0 resection margins in hepatoblastoma.

**Material and methods:**
This study was conducted according to PRISMA guidelines. A comprehensive literature search was performed in PubMed, Embase, and Web of Science, resulting in 957 studies, of which 6 were included in the analysis. Eligible studies included pediatric patients with hepatoblastoma undergoing surgical resection with reported resection margin status (R0 vs R1) and available data on overall survival (OS) and/or event-free survival (EFS). Reported hazard ratios (HRs) were extracted; when not available, HRs were reconstructed from published Kaplan–Meier curves using established digitization and reconstruction methods. Study-specific log(HR) and standard errors were pooled in random-effects models using restricted maximum likelihood estimation with Hartung–Knapp adjustment. Fixed-effect models were conducted as sensitivity analyses. Heterogeneity was quantified with $I^2$ and $\tau^2$ statistics.

**Results:**
Six studies comprising 1,418 patients (1,172 R0 and 246 R1 resections) met the inclusion criteria. For OS, the pooled HR for R1 versus R0 was 1.27 (95% CI, 0.93–1.72; $p = 0.10$), with no evidence of heterogeneity ($I^2 = 0\%$). For EFS, the pooled HR was 1.24 (95% CI, 0.64–2.41; $p = 0.44$), with moderate heterogeneity ($I^2 = 54\%$). Leave-one-out analyses did not materially change the results. Across both outcomes, there was no statistically significant difference in survival between R1 and R0 resections.

**Conclusion:**
This meta-analysis suggests that microscopically positive (R1) resection margins are not associated with significantly worse OS or EFS compared with R0 resections in hepatoblastoma. However, the current evidence is limited, and based on retrospective observational studies only. While R0 margins should generally be aimed for in hepatoblastoma surgery, the findings support the concept of R1 resection after neoadjuvant chemotherapy in locally advanced tumors involving major vascular structures. Consideration of R1 resection may therefore help avoid unnecessary transplantation in selected patients.

DE GRUYTER BRILL

**Impact of Time to Treatment Initiation on Survival in Pediatric Patients with Localized NRSTS**
ID: E-DCK-183

**Kinder- und Jugendchirurgie / Paediatric and Adolescent Surgery**
**Kinderchirurgische Forschung / Paediatric Surgery Research**

Illya Martynov[1], Jörg Fuchs[2], Guido Seitz[1]

[1]Universitätsklinikum Gießen Marburg (UKGM), Marburg, Deutschland, [2]Universitätsklinikum Tübingen, Tübingen, Deutschland

**Background:**
Time to treatment initiation (TTI) has been proposed as a prognostic factor in various malignancies, but data for pediatric non-rhabdomyosarcoma soft tissue sarcomas (NRSTS) are limited. We assessed the association between TTI and survival in children and adolescents or young adults (AYA) with localized NRSTS.

**Material and methods:**
We analyzed patients with localized NRSTS enrolled in two prospective clinical trials and one registry (CWS 96, CWS 2002P, SoTiSaR). TTI was defined as the interval between the date of biopsy or resection that established the histological diagnosis and the start of first sarcoma-specific therapy. Patients were stratified into five predefined subcohorts: Cohort A (biopsy followed by upfront resection, n = 208), Cohort B (pretreatment re-excision after initial resection, n = 204), Cohort C (upfront resection followed by adjuvant chemotherapy, n = 346), Cohort D (neoadjuvant chemotherapy following biopsy, n = 288), and Cohort E (non-resectable disease treated with chemotherapy, n = 132). Associations with event-free survival (EFS) and overall survival (OS) were assessed using multivariable Cox regression.

**Results:**
The pooled NRSTS cohort comprised 1178 patients, with a mean TTI of 26.8 days (range 1 to 114). In Cohort A (mean TTI 27.6 days), longer TTI was independently associated with inferior EFS (adjusted HR per day 1.016, 95% CI 1.005 to 1.028, $p = 0.005$), but not OS. A data-driven cutoff at 43 days identified significantly worse 5-year EFS for patients with TTI > 43 days (adjusted HR 2.58, 95% CI 1.41 to 4.72, $p = 0.002$). No significant association between TTI and survival was observed in Cohorts B to E.

**Conclusion:**
Delayed treatment initiation following diagnostic biopsy is independently associated with worse EFS in patients with localized resectable NRSTS and underscores the importance of timely surgical management.

DE GRUYTER BRILL

**Inflammation and Angiogenesis by Neutrophil Extracellular Traps in Murine Lungs is Tempered by DNase**
ID: E-DCK-242

**Kinder- und Jugendchirurgie / Paediatric and Adolescent Surgery**
**Kinderchirurgische Forschung / Paediatric Surgery Research**

Hong Zhang[1], Michael Boettcher[1], Julia Elrod[1], Michaela Klinke-Petrowsky[1], Jasmin Knopf[1], Richard Martel[1]

[1]Medizinische Fakultät Mannheim der Universität Heidelberg, Universitätsmedizin Mannheim, Klinik für Kinder- und Jugendchirurgie, Mannheim, Deutschland

**Background:**
The systemic inflammation during ECMO therapy activates neutrophils to form Neutrophil Extracellular Traps (NETs). On the one hand, NETs play a key role in the formation of inflammatory thrombi, which account for a significant proportion of complications during ECMO in newborns. Therefore, the therapeutic inhibition of NET formation is a point of application to prevent embolic events during ECMO, especially in newborns. On the other hand, NETs contain a proliferative effect in neoangiogenesis which is a desired process in the treatment of congenital malformations of the lung.
This study aimed to evaluate the modulating effect of the anti-NET substance DNase1 on neoangiogenesis and NET-induced inflammation in a murine model.

**Material and methods:**
Murine lungs in an explant culture were exposed to phorbol myristate acetate (PMA) activated neutrophils. Neoangiogenesis and inflammation were screened by a multiplex protein immunoassay (PEA) and the topological relationship between inflammation and neoangiogenesis was examined by immunofluorescence and quantified by digital image processing. DNase1 was added to inhibit potential NET-mediated effects.

**Results:**
PEA showed a reliable increase of several cytokines after exposure to activated neutrophils. Immunofluorescence revealed a colocalization between areas with increased cytokine levels and signs of angiogenesis. Both effects were diminished by the addition of DNase1 to the culture medium.

**Conclusion:**
Experimentally induced inflammation and angiogenesis are collocated when murine lung tissue is exposed to activated neutrophils. Therapeutic inhibition of NET formation inhibits NET-mediated inflammation and NET-induced expression of angiogenetic factors in murine lung tissue. Further translational studies should be conducted to investigate NET-inhibition as a promising adjunct to ECMO therapy with special consideration to neoangiogenesis.

DE GRUYTER BRILL

**Assessment of Inflammatory Parameters in Experimental Hepatoblastoma using Open Flow Microperfusion**
ID: E-DCK-256

**Kinder- und Jugendchirurgie / Paediatric and Adolescent Surgery**
**Kinderchirurgische Forschung / Paediatric Surgery Research**

Georg Singer[1], Sabine Obermüller[2], Bettina Leber[3], Hesham Elsayed[1], Günter Rauter[4], Thomas Altendorfer-Kroath[2], Holger Till[1], Beate Obermüller[1]

[1]Medical University of Graz, Department of Pediatric and Adolescent Surgery, Graz, Österreich, [2]JOANNEUM RESEARCH Forschungsgesellschaft mbH, HEALTH – Institute for Biomedical Research and Technologies, Graz, Österreich, [3]Medizinische Universität Graz, Klinische Abteilung für Allgemein-, Viszeral- und Transplantationschirurgie, GRaz, Österreich, [4]Medizinische Universität Graz, Department of Biomedical Research, Graz, Österreich

**Background:**
*In vivo* assessment of the tumor microenvironment (TME) in experimental hepatoblastoma (HB) remains unexplored. This study aimed to 1) develop an open-flow microperfusion (OFM) model for assessing the *in vivo* metabolic activity of the TME and 2) investigate potential differences in pro-inflammatory murine and human parameters in a murine HB model.

**Material and methods:**
Twenty-one BALB/c Fox1nu mice were subcutaneously injected with HuH6 cells (tumor group, n=15) or medium (control group, n=6). Following tumor growth, OFM catheters were inserted into both the HB and a liver lobe for comparison. After 4 hours of sampling, multiplex ELISAs were used to measure human inflammatory markers. Additionally, serum was taken to analyze murine and human inflammatory markers.

**Results:**
Twelve of 15 mice (80%) developed tumors with a mean weight of 1.16 g ± 0.47 g. Analysis of the serum inflammatory murine markers revealed significantly higher values of IL-6, IFN-β, G-CSF and MCP-1 in the tumor animals compared to controls. Six out of 27 measured human inflammatory parameters were detectable in the serum with significant differences between the groups for G-CSF, BLC, BTLA and CD80. Using OFM to assess the *in vivo* pro-inflammatory characteristics of HB, 27 parameters were detectable in both the tumor and the hepatic catheter fluid with significantly higher values of IL-4, IL-10, IL-12, MIP-2α, MIP-3β, TIM-3, caspase 3, BTLA and CD80 in the tumor.

**Conclusion:**
This study is the first to apply OFM in an experimental HB model, enabling real-time *in vivo* assessment of the tumor's metabolic activity. Our findings demonstrate significant inflammatory differences between the tumor and normal tissues, paving the way for future studies investigating intrahepatic HB inoculation using OFM as a more physiologically relevant approach.

Abstracts – DCK 2026 – 143rd Congress of the German Society of Surgery – Leipzig, April 22–24 • DOI 10.1515/iss-2026-2003
Innov Surg Sci 2026; 11 (Special Suppl 1): s270–s329

DE GRUYTER BRILL

**Learning Curve and Surgical Outcomes in Open Repair of CDH: A 23-Year Single-Center Experience**
ID: E-DCK-263

**Kinder- und Jugendchirurgie / Paediatric and Adolescent Surgery**
**Kinderchirurgische Forschung / Paediatric Surgery Research**

Kaja Riebesell[1]

[1]Kinder- und Jugendchirurgie UMM Mannheim, Mannheim, Deutschland

**Background:**
Operative repair of congenital diaphragmatic hernia (CDH) is a technically demanding procedure. The impact of a surgeon’s training level and cumulative case load on postoperative outcomes remains insufficiently defined. This study aims to evaluate the influence of surgical training level and individual experience on complications and recurrence rates following open CDH repair.

**Material and methods:**
A single-center analysis of all patients undergoing open CDH repair over a 23-year period was performed. The follow up was at least 2-years. Surgeons were stratified by years of surgical training and by the number of individually performed CDH repairs. Primary endpoints were operative duration, complication rates, and recurrence rates. In a subgroup of surgeons with ≥20 open CDH repairs, the first 10 procedures were compared with the last 10 cases per surgeon.

**Results:**
In total, n patients were identified, of whom 1011 met the inclusion criteria. Complication and recurrence rates did not differ significantly across training levels. Similarly, among experienced surgeons, outcomes were comparable between early and later cases. In contrast, operative duration decreased significantly with growing individual experience.

**Conclusion:**
Open repair of CDH can be performed safely by surgeons at different stages of training without compromising complication or recurrence rates, provided that procedures are supervised by senior attendings. While operative efficiency improves with accumulating experience, patient outcomes remain stable across training levels. These findings underscore the importance of structured training pathways and effective supervision, supporting the integration of junior surgeons into high-complexity index procedures while maintaining consistent standards of care in pediatric surgery.

DE GRUYTER BRILL

**Digging Deeper: mRNA Isoform Analysis identifies mucosal Differences in Hirschsprung's Disease.**
ID: E-DCK-288

**Kinder- und Jugendchirurgie / Paediatric and Adolescent Surgery**
**Kinderchirurgische Forschung / Paediatric Surgery Research**

Johanna Hagens[1], Malik Alawi[2], Konrad Reinshagen[1], Christian Tomuschat[1]

[1]University Medical Center Hamburg-Eppendorf, Department of Pediatric Surgery, Hamburg, Deutschland, [2]University Medical Center Hamburg-Eppendorf, Bioinformatics Core, Hamburg, Deutschland

**Background:**
Although Hirschsprung’s Disease has been studied over decades and possible predisposing genetic constellations have been described, its multifactorial genesis still hinders complete understanding of differences to non-Hirschsprung patients [1-3]. Hirschsprung’s Disease is associated with inflammatory complications such as the accompanying associated enterocolitis (HAEC), but the underlying pathology remains unclear [4,5]. Including the novel technique of mRNA isoform determination into our transcriptome data on organoids originated from Morbus Hirschsprung patients, we aimed to gain deeper insights in Hirschsprung’s mucosal pathophysiology.

**Material and methods:**
Adult stem cell organoids (AdSC) were derived from *n*=10 Hirschsprung patients (HSCR) and *n*=10 non-Hirschsprung controls (nHSCR). Organoids were stimulated with proinflammatory cytokines TNF-alpha, Interleukin-1 beta and Interleukin-6 to induce proinflammatory stress to simulate Hirschsprung’s associated enterocolitis. Differential gene expression was conducted in whole mRNA transcriptome analysis (TA). Genes with significant mRNA isoform divergency were identified using differential isoform expression analysis (IEA).

**Results:**
TA detected *n*=163 (*n*=95 up; *n*=68 down) differentially expressed genes (DEGs) between the two groups, mainly addressing pathways for cytokine signaling and inflammation before influence of stimulation, but only *n*=21 (*n*=13 up; *n*=8 down) after. IEA identified *n*=61 genes with divergent isoform expression (GDIs). Only *n*=3 GDIs were identical with DEGs found in TA. *n*=6 GDIs matched the combined DIF cut-off >0.45. Molecular functions of these targets included DNA repair, cellular stress response, cell survival, and innate and bacterial immune response. GDIs were reported to be mainly expressed in endothelial and enteric glia cells for colonic tissue.

**Conclusion:**
Whole mRNA expression showed differences between HSCR and nHSCR mainly in inflammatory signaling targets before a cytokine influence, indicating a preexisting aberrant genetic profile. We for the first time were able to identify various additional genes with divergent mRNA transcripts addressing cellular damage response and immunology, overlooked by whole mRNA transcriptome alone. Although impact on further protein translation remains unclear, these findings open the door for the understanding of Hirschsprung's Disease on a deeper genetic level.

[1] Tilghman JM, Ling AY, Turner TN, Sosa MX, Krumm N, Chatterjee S, Kapoor A, Coe BP, Nguyen KH, Gupta N, Gabriel S, Eichler EE, Berrios C, Chakravarti A. *Molecular Genetic Anatomy and Risk Profile of Hirschsprung's Disease.* **N Engl J Med**. 2019 Apr 11;380(15):1421-1432. doi: 10.1056/NEJMoa1706594. PMID: 30970187; PMCID: PMC6596298.

[2] Gunadi, Vujira KA, Amadeus VC, Gabriela GC, Amaragati AR, Geometri ET, Eryvinka LS, Ferdinandus KE, Pramono NA, Dwihantoro A. *Aberrant Expressions of EDNRB and EDN3 in a Multifactorial Hirschsprung Disease.* **Curr Pediatr Rev**. 2025 Jan 22. doi: 10.2174/0115733963343518241223193627. Epub ahead of print. PMID: 39844409.

[3] Karim A, Tang CS, Tam PK. *The Emerging Genetic Landscape of Hirschsprung Disease and Its Potential Clinical Applications*. **Front Pediatr**. 2021 Aug 5;9:638093. doi: 10.3389/fped.2021.638093. PMID: 34422713; PMCID: PMC8374333.

[4] Lewit RA, Kuruvilla KP, Fu M, Gosain A. *Current understanding of Hirschsprung-associated enterocolitis: Pathogenesis, diagnosis and treatment*. **Semin Pediatr Surg**. 2022 Apr;31(2):151162. doi: 10.1016/j.sempedsurg.2022.151162. Epub 2022 Apr 8. PMID: 35690459; PMCID: PMC9523686.

[5] Li S, Zhang Y, Li K, Liu Y, Chi S, Wang Y, Tang S. *Update on the Pathogenesis of the Hirschsprung-Associated Enterocolitis*. **Int J Mol Sci**. 2023 Feb 27;24(5):4602. doi: 10.3390/ijms24054602. PMID: 36902033; PMCID: PMC10003052.

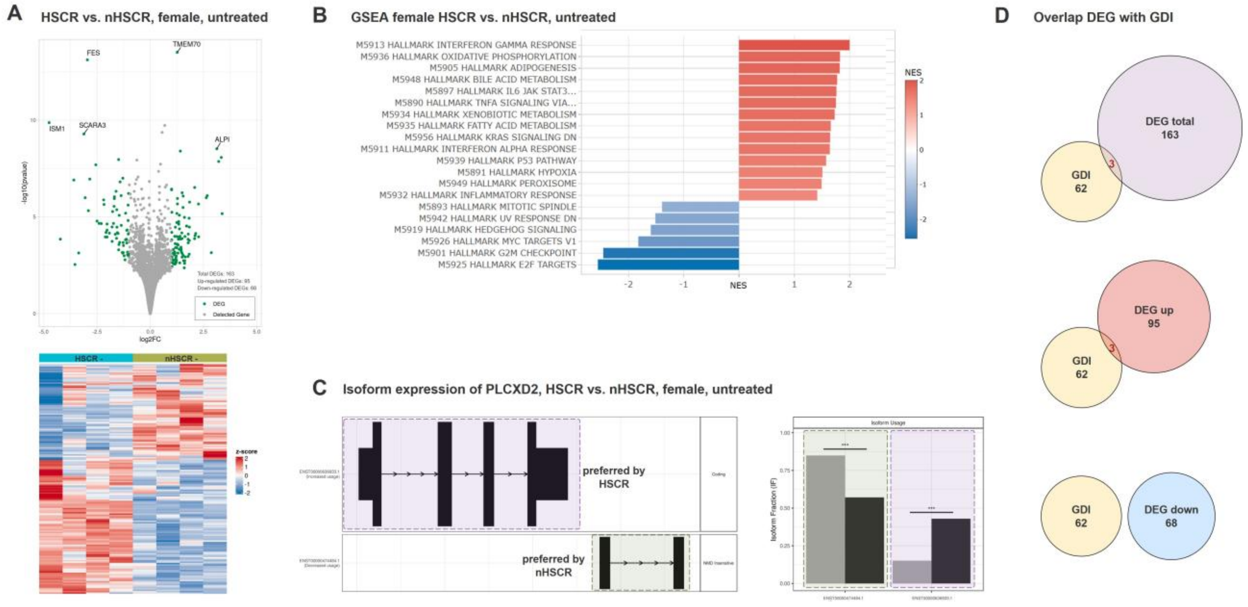

DE GRUYTER BRILL

**Clinical Course of Recurrence in Pediatric Perforated Appendicitis After Non-Operative Care**
ID: E-DCK-303

**Kinder- und Jugendchirurgie / Paediatric and Adolescent Surgery**
**Kinderchirurgische Forschung / Paediatric Surgery Research**

Vincent Rohrbacher[1], Richard Gnatzy[1], Jan-Hendrik Gosemann[1], Martin Lacher[1], Richard Wagner[1]

[1]Klinik und Poliklinik für Kinderchirurgie - Uniklinik Leipzig, Leipzig, Deutschland

**Background:**
Conservative management in select cases of perforated pediatric appendicitis with intravenous antibiotics ± abscess drainage is well established. The necessity of routine interval appendectomy remains controversial. Reported recurrence rates range from 10–40%, but detailed data on the clinical course and complications of recurrences are scarce. This study aimed to analyze recurrence episodes more detailed and provide evidence for clinical decision-making after initially non-operative management.

**Material and methods:**
We retrospectively analyzed children treated non-operatively for perforated appendicitis at Leipzig University Hospital during an 8-year period (2016-2024). Among those all children treated non-operatively for perforated appendicitis ± abscess and/or RLQ palpable mass were identified and analyzed. Data included demographics, clinical parameters (length of stay, recurrence of appendicitis ± abscess, interval appendectomy), laboratory values (white blood cell count, C-reactive protein at admission/discharge), radiological findings (abscess size, appendicolith, abdominal mass), microbiological results, and management (antibiotic regimens, drainages, ICU treatment). The follow-up period was up to nine years

**Results:**
Among 675 patients with perforated appendicitis, 70 (10,4%; 30 girls, mean age 7.5 years [1.7-17.4 years]) underwent initial non-operative management. At discharge, none of the patients had signs of abscess or ongoing infection. Recurrence occurred in 20 patients (28.5%), with a median of 20 days after discharge [1–1770 days]. Of these, 13 children (65%) developed another episode of intra-abdominal abscess. Amongst these, drainage was required in 43.8% of recurrent cases (vs. 57.3% of cases at initial course). Compared with the initial episode, recurrences had milder clinical presentation: mean WBC 17.5/nl vs. 15.5/nl, mean CRP 158 mg/l vs. 75.9 mg/l, median hospital stay 12 vs. 6.5 days, median duration of intravenous antibiotics 11.3 vs. 6.4 days, and median drainage duration 8.5 vs. 6.6 days. Interval appendectomy was performed in 51 patients (75.4%), with a median of 67.5 days after initial discharge. Their median postoperative hospital stay was 3 days.

**Conclusion:**
Recurrence after non-operative management of perforated appendicitis in children is associated with significant morbidity, particularly from abscess formation. This risk should be considered when discussing prognosis with parents and taken into account in decisions regarding interval appendectomy.

DE GRUYTER BRILL

**Troponin and NT-proBNP as Indicators for Ischemic Colitis in Patients with Congenital Heart Disease**
ID: E-DCK-331

**Kinder- und Jugendchirurgie / Paediatric and Adolescent Surgery**
**Kinderchirurgische Forschung / Paediatric Surgery Research**

Kristine Dräger[1], Nariman Mokhaberi[2], Philipp Schneider[3], Ida Hüners[3], Daniel Biermann[3], Michael Hübler[3], Sebastian Harms[3], Omid Madadi-Sanjani[1], Christian Tomuschat[1], Konrad Reinshagen[1 2]

[1]UKE, Kinderchirurgie, Hamburg, Deutschland, [2]AKK, Kinderchirurgie, Hamburg, Deutschland, [3]Universitäres Herz- und Gefäßzentrum, Hamburg, Deutschland

**Background:**
Ischemic colitis (IC) in children with congenital heart disease (CHD) is a rare but serious complication that occurs primarily in children with complex cardiac defects. Patients with CHD have impaired cardiac output, which can lead to decreased oxygenation and insufficient blood flow to the intestinal wall. This hypoperfusion significantly increases the risk of intestinal injury and, in severe cases, the need for bowel resection[1].
Choosing the right time for surgery is particularly challenging. The clinical presentation is often nonspecific, and imaging cannot always reliably detect the extent of ischemia. Surgery performed too early carries the risk of unnecessary resections, while surgery performed too late worsens the prognosis due to progressive sepsis and multiple organ failure[2].
The present study aims to investigate whether cardiac biomarkers, specifically Troponin I and N-terminal pro B-type natriuretic peptide (NT-proBNP), can serve as early indicators for the development of ischemic colitis in pediatric CHD patients. By identifying potential predictive markers, this study seeks to contribute to earlier diagnosis and potentially better outcomes for this vulnerable patient group.

**Material and methods:**
This study was designed as a retrospective cohort analysis including pediatric patients diagnosed with congenital heart disease (CHD) who developed ischemic colitis (IC) and who underwent surgery at the Department of Pediatric Surgery at the University Medical Center Hamburg-Eppendorf (UKE) between January 2013 and May 2023, either before or after cardiac surgery or intervention (IC-CHD, n=21). The control group consisted of all patients with CHD who underwent cardiac surgery or intervention in 2023 before the age of 18 months but did not develop surgically treated IC (CHD, n=109).
Levels of Troponin I and proBNP were recorded for all patients in the interval 8 days before and after cardiac surgery or intervention, whereby values were not available for all patients every day.
For each group and day, mean values were calculated and presented using descriptive statistics. All statistical analyses were conducted using GraphPad Prism version 10.0.0.

**Results:**
A total of 130 pediatric patients with congenital heart disease (CHD) were included in this study, of whom 21 developed ischemic colitis (IC-CHD group) and 109 did not (CHD group).

Troponin I levels were comparable between both groups. However, a marked difference was observed on postoperative day 1, with significantly higher levels in the CHD group (14.924 pg/ml) compared to the IC-CHD group (6.740 pg/ml).
NT-proBNP levels were generally higher in the IC-CHD group, particularly from one day preoperatively to one day postoperatively. On postoperative day 5, NT-proBNP levels showed a relative increase in the IC-CHD group, reaching a mean of 41.890 ng/ml, which appeared higher than the corresponding value in the CHD group (14.217 ng/ml).
On average, bowel surgery in the IC-CHD group was performed 8 days after the initial cardiac surgery or intervention.

**Conclusion:**
This study provides initial indications of differences between the two groups and may serve as a basis for further hypothesis-building analysis. Elevated Troponin I levels at the time of cardiac surgery or intervention do not appear to indicate the development of ischemic colitis or the need for subsequent bowel surgery. In fact, patients with the highest Troponin I levels did not develop ischemic colitis. Conversely, patients who developed ischemic colitis showed markedly elevated NT-proBNP levels on postoperative or postinterventional day 5 and underwent bowel surgery on average three days later. Elevated NT-proBNP levels may indicate worsening systemic strain, which precedes surgery, but specificity remains unclear.
As this analysis is based on descriptive statistics, further evaluation is planned by including additional patients from the years 2024 and 2025 to improve statistical significance. Moreover, future analyses will incorporate variables such as birth weight, prematurity, and severity of the CHD to allow for more nuanced group-specific conclusions.

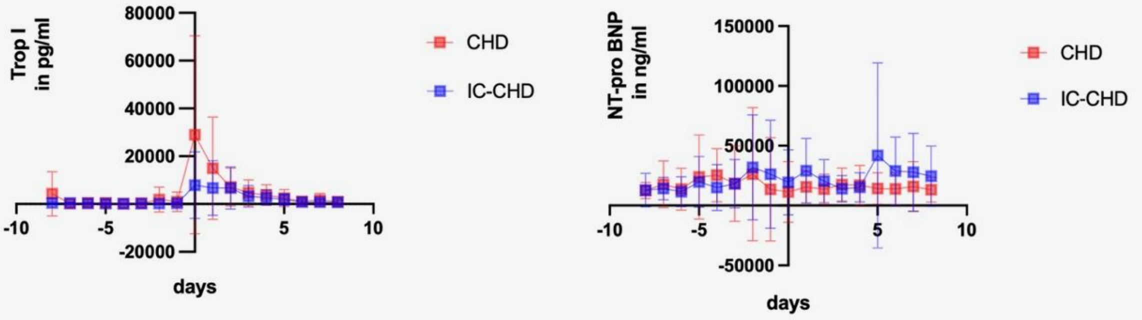

Fig. 1: mean values (± standard deviation) of Troponin I and NT-pro BNP eight days before and after cardiac surgery or intervention.

DE GRUYTER BRILL

**Exposure to pesticides in drinking water during pregnancy and onset of congenital malformations**
ID: E-DCK-332

**Kinder- und Jugendchirurgie / Paediatric and Adolescent Surgery**
**Kinderchirurgische Forschung / Paediatric Surgery Research**

Cristiana Riboni[1], Chiara Paganetti[1], Stephanie Gros[1]

[1]UKBB, Basel, Schweiz

**Background:**
Congenital malformations (CM) are the leading cause of infant mortality. The aetiology remains unknown in 70% of cases. The most accepted hypothesis is that hereditary and environmental elements concur in altering embryo-foetal development. Recently, the role of the environment has been emphasized.
**Objectives**: Women are exposed to several xenobiotics during pregnancy. This review aims to study the available literature about the exposure of pregnant women to pesticides through drinking water to see if there is any evidence of correlation to the onset of any kind of congenital anomalies.

**Material and methods:**
We conducted a systematic literature review in The Cochrane Library, Embase and PubMed for study published from January 1, 2005 and April 21, 2025. Article which have as population pregnant women, as exposure toxins present in drinking water and as main outcome any type of congenital anomalies were included. The screening of title, abstract and full text as well as the data extraction was conducted independently thorough two investigators. A third investigator resolved conflicts. Each included study was evaluated according to the NIH's quality assessment tools. GRADE approach was used for summarizing and assess certainty in the bodies of evidence produced by the review. This study is registered with PROSPERO, CRD420251063011.

**Results:**
In recent years, the role of the environment as an important element of reproductive risk has been emphasized, and this aspect has received increasing interest. Pregnant women are particularly susceptible to xenobiotics, particularly in the first trimester, and it is estimated that during pregnancy they can come in contact with around 160 different chemicals through food and drinking water. While many agricultural pesticides with potential teratogenic effects, such as nitrofen itself, have been discontinued, instances of harmful substance transgressions in drinking water continue to be reported. Among this substances pesticides most exceed the limits in ground water. These factors can interfere with normal organogenesis through preconception mutagenic action, postconceptional teratogenic action, endocrine interference action, and epigenetic action.
Glyphosate seems to have an effect on endocrine and reproductive function in humans.
Periconceptional maternal exposure to atrazine has been correlated to hypospadias and cryptorchidism, as well as to abdominal wall defect, such as gastroschisis, neural tube defect, cleft lip, choanal atresia, limb and hearth defect.
Organochlorine pesticides has been shown to be a risk factor for Neural tube defects.

**Conclusion:**

Literature about exposure to toxins through drinking water is scarce and the results are inconclusive. However, several studies pointed out a possible correlation between toxins detected in drinking water and the onset of congenital malformation. Further studies with direct measurements of exposure need to be done to better understand the aetiology of malformation. The knowledge gained could guide inform campaigns for pregnant women, contributing the prevention of the disease.

Abstracts – DCK 2026 – 143rd Congress of the German Society of Surgery – Leipzig, April 22–24 • DOI 10.1515/iss-2026-2003
Innov Surg Sci 2026; 11 (Special Suppl 1): s270–s329

**High-fidelity Model For Surgical Simulation Of Laparoscopic Common Bile Duct Exploration (LCBDE)**
ID: E-DCK-339

**Kinder- und Jugendchirurgie / Paediatric and Adolescent Surgery**
**Kinderchirurgische Forschung / Paediatric Surgery Research**

Tobias Jhala[1], Lukas Bischoff[1], Sabine Zundel[2], Christoph Slavetinsky[1], Jonathan Aichner[3], Maximiliano Maricic[4], Jörg Fuchs[1], Hanna Alemayehu[5], Luke Neff[6]

[1]Universitätsklinikum Tübingen, Radiologie, Tübingen, Deutschland, [2]Kantonsspital Luzern, Luzern, Schweiz, [3]Great Ormond Street Hospital, London, Großbritannien, [4]Hospital de Pediatría Dr. J.P. Garrahan, Buenos Aires, Argentina, [5]Emory University School of Medicine, Atlanta, Vereinigte Staaten, [6]Wake Forest University School of Medicine, Winston-Salem, Vereinigte Staaten

**Background:**
Gallstone disease is increasing across all age groups, and 10–20% of pediatric cholecystectomy patients present with choledocholithiasis. In adults, guidelines recommend ERCP for bile duct clearance followed by laparoscopic cholecystectomy. However, ERCP in children carries higher complication rates, exposes patients to two general anesthetics, and still leaves residual or newly dislodged stones in up to 13% of cases at subsequent surgery. By contrast, a surgery-first approach—laparoscopic cholecystectomy with intraoperative cholangiogram and step-up to laparoscopic common bile duct exploration (LCBDE) if needed—achieves clearance rates comparable to ERCP while reducing complications, length of stay, and costs. Furthermore, total anesthetic exposure is not increased. Despite this, the renaissance of LCBDE is hindered by a steep learning curve and limited training exposure. High-fidelity simulation models are therefore essential, but current trainers lack sufficient realism for critical steps such as wire manipulation and balloon sphincteroplasty.

**Material and methods:**
We developed a low-cost, high-fidelity simulation model using cadaveric chicken hearts and great vessels to replicate extrahepatic biliary anatomy. The right innominate artery represented the common bile duct, the subclavian artery the cystic duct, the carotid artery the hepatic duct, and the aortic valve the sphincter of Oddi. Artificial stones (peppercorns) were introduced, and staged clearance techniques—guidewire insertion, intraoperative cholangiography, forced flushing, and balloon dilatation—were performed with standard laparoscopic instruments.

**Results:**
The model reproduced key anatomical relationships and provided realistic haptic feedback. Transcystic access, wire guidance, and balloon sphincteroplasty could be practiced reliably. Fluoroscopy use enhanced training but was optional. Preparation was straightforward, reproducible, and inexpensive, making the model suitable for repeated use.

**Conclusion:**

This cadaveric chicken heart model offers a practical, high-fidelity simulation platform for LCBDE. It provides realistic anatomy and tactile conditions at low cost, overcoming limitations of current trainers. The model has potential to support skill acquisition, facilitate structured training, and contribute to the reintroduction of LCBDE into surgical practice. Validation and integration into curricula represent the next steps.

DE GRUYTER BRILL

**Defining Entrustable Professional Activities (EPAs) in Pediatric Surgery for German Medical Students**
ID: E-DCK-341

**Kinder- und Jugendchirurgie / Paediatric and Adolescent Surgery**
**Kinderchirurgische Forschung / Paediatric Surgery Research**

Alina Schacherl[1], Elisabeth Narciss[2], Katrin Schüttpelz-Brauns[3], Udo Obertacke[4], Anne Herrmann-Werner[5], Michael Boettcher[1], Marietta Jank[1]

[1]Department of Pediatric Surgery, University Medical Center Mannheim, University of Heidelberg, Mannheim, Germany., Mannheim, Deutschland, [2]Competence Center for final-year education, Medical Faculty Mannheim, Heidelberg University, Mannheim, Germany., Mannheim, Deutschland, [3]Department of Medical Education Research, Medical Faculty Mannheim, Heidelberg University, Mannheim, Germany., Mannheim, Deutschland, [4]Department of Orthopaedic and Trauma Surgery, University Medical Centre Mannheim, Medical Faculty Mannheim, University of Heidelberg, Mannheim, Germany., Mannheim, Deutschland, [5]TIME -Tübingen Institute for Medical Education, Medical Faculty, University of Tuebingen, Tuebingen, Germany., Tübingen, Deutschland

**Background:**
The final-year clinical clerkship in Germany (German: Praktisches Jahr; short: PJ) marks the transition from medical school to residency requiring growing expectations on clinical responsibility. Entrustable Professional Activities (EPAs) provide a competency-based framework to define and assess core clinical tasks. We aim to identify relevant EPAs in pediatric surgery to promote standardized and practice-oriented training for medical students across Germany.

**Material and methods:**
We first assessed current PJ requirements in pediatric surgery across Germany. A nationwide survey of pediatric surgery departments collected data on (1) whether pediatric surgery is offered as a stand-alone or integrated rotation, (2) the number of PJ students per rotation, (3) the presence of physician assistants and (4) the availability of affiliated teaching hospitals. In parallel, we analyzed existing student logbooks, examining structure, scope and listed competencies.

**Results:**
Department heads and teaching coordinators from 39 medical faculties (94.9% public, 5.1% private) were contacted and 27 surveys (69.2%) have been completed to date. 44.4% (n=10) of medical schools offer pediatric surgery as part of general surgical or pediatric rotations, while 55.6% (n=15) provide it as a dedicated rotation. Among the latter, 46.7% (n=7) also offer an additional placement within the surgery part of the PJ. The number of medical students per rotation ranged from 1 to 4, irrespective of the size of the pediatric surgical team. 64% (n=16) of logbooks were specific to pediatric surgery. The remaining 36% (n = 9) used the general surgery logbook. Currently, 25 logbooks are available for analysis, and data from 11 logbooks have been integrated into a longlist of competencies. 421 competencies have been extracted from 11 logbooks. The most frequently required competencies for medical students are physical examination, documentation, postoperative management and taking medical history. Full analysis will be completed by the time of the conference. The longlist will be refined by a threshold to generate a shortlist of the most frequent competencies. This will provide the basis for a Delphi process involving pediatric surgeons, PJ students, and medical education experts.

**Conclusion:**
Our findings highlight the variability in pediatric surgery training during the PJ and underscore the need for clearly defined EPAs. By aligning expected competencies with those of a first-year resident, a revised logbook will offer PJ students a structured framework for skill acquisition and ensure a smoother transition into residency.

DE GRUYTER BRILL

**Quality of Online Patient Information on Biliary Atresia: An Expanded EQIP Assessment**
ID: E-DCK-361

**Kinder- und Jugendchirurgie / Paediatric and Adolescent Surgery**
**Kinderchirurgische Forschung / Paediatric Surgery Research**

Sofia Ahrens[1], Omid Madadi-Sanjani[2], Marie Uecker[2], Christian Tomuschat[2], Konrad Reinshagen[3], Nariman Mokhaberi[3]

[1]Altonaer Kinderkrankenhaus, Hamburg, Deutschland, [2]Universitätsklinikum Hamburg-Eppendorf, Hamburg, Deutschland, [3]Universitätsklinikum Hamburg-Eppendorf, Altonaer Kinderkrankenhaus, Hamburg, Deutschland

**Background:**
Biliary atresia (BA) is a rare condition in neonates, defined by the progressive obliteration of extra- and intrahepatic bile ducts. In the absence of intervention, BA leads to hepatic degeneration and cirrhosis. The primary therapeutic intervention is the Kasai hepatoportoenterostomy (KPE). Nevertheless, only about 20–30 % of patients survive long-term with their own liver and therefore require a liver transplant during the course of their illness.
In circumstances where grave conditions are communicated by medical practitioners, families are frequently overwhelmed and unable to process the information. In these cases, the internet can be a valuable source of additional information, helping families to understand the disease, its implications, and their child's future medical needs. The objective of this study is therefore to evaluate the quality of information about BA that is available online.

**Material and methods:**
The two search terms “biliary atresia” and “kasai surgery” were used to conduct an internet search across the three most popular search engines (Google, Yahoo, Bing). The data selection was performed between June and September 2025 and the “geolocation” setting was turned off in order to receive location independent search results. The first 100 websites from each search were screened and classified. The following predefined exclusion criteria were used to identify and remove irrelevant websites: scientific articles, websites targeted at medical practitioners, websites with restricted access, websites in other languages than English, links to videos and duplicates. The final results were then evaluated independently by two physicians using the expanded version of the ensuring quality information for patients (EQIP) scale.

**Results:**
Following the application of the exclusion criteria to the initial search result of 600 websites, a final total of 75 websites were retained for further analysis. 63 % of these websites were from the USA, with private hospitals being the most frequent ones. The overall median EQIP score was 47.1 %, with only three websites demonstrating EQIP scores above 70 %. A mere 35% of websites were found to report on quantitative estimates of risks of BA and KPE. It was shown that information concerning the immediate pre- and post-operative course was documented in only 10.7 % and 26.7 % of cases, respectively. Furthermore, a bibliography of used evidence-based data (25 %) or further sources of reliable information or support (23 %) were scarcely provided. The recommended identification data, including the date of issue (48 %), the name of the persons or entities who produced (27 %) or financed (3 %) the document, was not included on a regular basis. Only 24 % of websites provided illustrations or diagrams.

**Conclusion:**
The results of our study demonstrate considerable gaps of high-quality patient information on BA on the internet. Only a small number of the websites comprehensively addressed all pertinent aspects, such as the immediate pre- and post-operative course, the long-term course after KPE and the potential complications associated with BA and their corresponding therapies. These results underscore the pressing need for readily accessible and comprehensive high-quality information resources concerning BA on the internet. References, information regarding the document's creator, and other reliable informational materials should be provided in the context of transparent documentation.

DE GRUYTER BRILL

**Mobile Health Applications in Pediatric Surgery: Systematic App Store and Literature Review**
ID: E-DCK-398

**Kinder- und Jugendchirurgie / Paediatric and Adolescent Surgery**
**Kinderchirurgische Forschung / Paediatric Surgery Research**

Tessa Hermann[1], Charlotte Reich[1], Cui Yang[2], Preetha Moorthy[3], Michael Boettcher[1], Marietta Jank[1]

[1]Klinik für Kinder- und Jugendchirurgie Mannheim, Mannheim, Deutschland, [2]Medizinische Fakultät Mannheim, Mannheim, Deutschland, [3]Department of Biomedical Informatics at the Center for Preventive Medicine and Digital Health, Mannheim, Deutschland

**Background:**
Mobile health (mHealth) applications are increasingly used in clinical practice. In pediatric surgery, they may improve perioperative care, patient engagement and family support. We aim to summarize current literature and available smartphone applications targeting children with pediatric surgical conditions and their caregivers.

**Material and methods:**
The Apple App Store and Google Play Store were systematically searched using medical terms and lay expressions that parents and children/teenagers might use (e.g., “child” AND “surgery”, “wound”, “birth defect”, “follow up”, “pediatric surgery”)Apps were eligible if they were patient- or family-directed, available in English or German and accessible on iOS or Android. Expert evaluation with the MARS-G is ongoing. In parallel, a systematic literature review was conducted across PubMed, Embase, Cochrane Library, Web of Science, and Google Scholar. We included studies on mHealth apps for pediatric surgical patients (≤18 years) and their families. Exclusion criteria comprised non-surgical conditions, adult populations or interventions not delivered via smartphone or tablet. Two reviewers independently performed screening and data extraction.

**Results:**
The app store search identified 1,561 applications (376 iOS, 1,185 Android). After screening, 11 apps met the inclusion criteria: liver transplantation (n=2), cancer (n=3), bladder exstrophy (n=1), clubfoot (n=1), pain assessment (n=2), and medical language simplification (n=2). Functionalities included education (n=9), symptom tracking (n=2), communication (n=2), gamification (n=3) and treatment tracking (n=1). Most were iOS-only (n=7, 78%), while two were available for Android (n=2, 22%); only two apps (22%) were accessible on both platforms. All were free of charge. No user reviews were available. Preliminary MARS-G ratings showed consistent scoring for engagement, functionality and information quality, with variability in aesthetics.
The literature search yielded 4,079 records; 94 full texts are currently under review for study characteristics, app features and clinical outcomes. Data extraction will be completed by the time of the conference.

**Conclusion:**

Few mHealth apps have been developed for pediatric surgery and those available lack clinical validation and user feedback in app stores. Most focus primarily on patient education rather than outcome-driven features. These findings underscore the need for evidence-based, co-designed applications that are rigorously evaluated and meaningfully integrated into pediatric surgical care pathways.

DE GRUYTER BRILL

**Congenital esophageal stenosis related to tracheobronchial remnant: balloon dilation vs. resection**
ID: E-DCK-407

**Kinder- und Jugendchirurgie / Paediatric and Adolescent Surgery**
**Kinderchirurgische Forschung / Paediatric Surgery Research**

Miroslav Koychev[1], Oliver Muensterer[1]

[1]Kinderchirurgische Klinik und Poliklinik im Dr. von Haunerschen Kinderspital, München, Deutschland

**Background:**
Congenital esophageal stenosis (CES) related to tracheobronchial remnant (TBR) is an extremely rare malformation, typically diagnosed in infants and young children. Endoscopic dilatation or resection and anastomosis are both accepted therapeutic options, but the superiority of one versus the other remains controversial.

**Material and methods:**
We performed a comprehensive literature search in PubMed and MEDLINE using standardized search terms and subject headings from 1969 onwards. Inclusion criteria were patient age 0 to 18 years, confirmed diagnosis of CES due to TBR, English language, intervention by balloon dilatation or surgical resection, and reporting of outcome.

**Results:**
A total of 215 articles were identified, of which 33 met the inclusion criteria. These consisted of 11 retrospective observational studies, 10 case series, and 12 single case reports. In total, 183 were included in our analysis, of which 103 were initially treated with resection/anastomosis, and 80 with primary dilatation. Patients who underwent primary resection required between 1 and 3 additional dilations. Patients who underwent initial dilatation required between 1 and 7 re-dilations. The resection/anastomosis group group demonstrated a significantly lower mean complication rate (0.2%) compared to those who underwent primary dilatation (13.8%, $p<0.01$).

**Conclusion:**
Both resection and balloon dilatation are viable options to treat CES associated with TBR. While those undergoing upfront dilatation are more likely to require further interventions, some may be spared a more invasive operative procedure.

DE GRUYTER BRILL

**JJ stent removal in pediatric patients by Trans urethral catheterization technique: A clinical trial**
ID: E-DCK-409

**Kinder- und Jugendchirurgie / Paediatric and Adolescent Surgery**
**Kinderchirurgische Forschung / Paediatric Surgery Research**

Roohullah Hares[1]

[1]French Medical Institute for Mothers and Children (FMIC), Department of Pediatric Surgery, Kabul, Afghanistan

**Background:**
JJ stents placed at the end of pediatric urological surgeries which require another cystoscopy under general anaesthesia for removal. We developed a novel technique for the first time for entrapping the JJ stent in outpatient department (OPD).
The aim of the study is to describe the novel technique for JJ stent removal and comparing of this technique with cystoscopy. Therefore, the study designed a randomized trials where the trans urethral catheterization technique evaluated against cystoscopy which is a gold standard for removal of JJ stents.

**Material and methods:**
A clinical trial carried out on 160 child patients, including 106 boys and 54 girls, who had a JJ ureteral stent were randomly assigned 1:1 to two groups, which were subjected to non-cystoscopic group and cystoscopic group respectively, at FMIC and Maiwand teaching hospital.
Patients were evaluated for success rate, costs, hospital length of stay and postoperative complications.

**Results:**
In this study, 160 children with JJ stents were enrolled from June 2019 to December 2021. They were randomised by equal allocation (1:1) to cystoscopic and trans urethral catheterization technique removal arms. The catheterization technique and cystoscopy were successful in 95% (76/80) and in 100% (80/80) cases, respectively, with no statistically significant difference in the outcome ($p = 0.182$). The average cost for cystoscopic removal of the stent was 17,859 Afs and was 1483 Afs for the catheterization technique.
Also the mean length of stay at hospital of non-cystoscopy group was significantly shorter than that of cystoscopy group (0.35±0.4 hour Vs 8.29±4 hour).
There was no significantly difference in complications between two groups.

**Conclusion:**
The trans urethral catheterization technique for JJ stents removal is a practical low-cost and less hospital length of stay safe alternative to cystoscopic removal of JJ stents in children. Although the technique has a high success rate, it still needs the backup option of cystoscopy under general anaesthesia.

DE GRUYTER BRILL

**Traction versus interposition in long-gap esophageal atresia – a retrospective study**
ID: E-DCK-423

**Kinder- und Jugendchirurgie / Paediatric and Adolescent Surgery**
**Kinderchirurgische Forschung / Paediatric Surgery Research**

Karin Grimps[1], Oliver Muensterer[1]

[1]Ludwig-Maximilians-Universität München, München, Deutschland

**Background:**
The management of long-gap esophageal atresia (LGEA) includes traction or interposition techniques to establish digestive tract continuity. The objective of this study is to compare the outcome of these alternatives in terms of feeding commencement and dilatations/stricture formation.

**Material and methods:**
Retrospective analysis was performed on all patients with LGEA, managed with either a traction or interposition technique over a 10 year period at 2 university medical centers. Outcome variables included hospital stay, time to oral feeds, stricture rate, and postoperative number of dilatations.

**Results:**
From 01/2012 through 06/2022, a total of 28 LGEA patients were included. In the interposition group (n=7), 6 patients underwent gastric pull-up and 1 patient jejunal interposition. In the traction group (n=21), 14 patients underwent a handsewn anastomosis (HA), and 7 patients magnetic compression anastomosis (MCA). A mean of 2.5±1.3 (n=21) traction procedures were performed prior to establishing definitive continuity. The average age at time of definitive surgery was 225±212 days. Oral feedings commenced at a mean of 18±8 days after interposition, 20±15 days after traction/MCA, and 34±17 days after traction/HA. There was no statistically significant difference in feeding commencement or hospital stay. There was a significant difference in terms of dilatations: the mean number of dilatations performed was 1.0±2.6 in the interposition group, 3.1±2.2 in the traction/MCA group and 5.9±6.5 in the traction/HA group, p=0,04 interposition vs. traction/MCA.

**Conclusion:**
While most outcome parameters were similar across all groups, traction techniques were associated with more dilatations than interposition.

DE GRUYTER BRILL

**Enhancing AI Readiness in Pediatric Surgery: Impact of a Workshop on Knowledge and Competencies**
ID: E-DCK-425

**Kinder- und Jugendchirurgie / Paediatric and Adolescent Surgery**
**Kinderchirurgische Forschung / Paediatric Surgery Research**

Georg Singer[1], Hesham Elsayed[1], Beate Obermüller[1], Tristan Till[2], Richard Gnatzy[3], Sebastian Tschauner[2], Holger Till[1]

[1]Medical University of Graz, Department of Pediatric and Adolescent Surgery, Graz, Österreich, [2]Medical University of Graz, Division of Pediatric Radiology, Department of Radiology, Graz, Österreich, [3]University of Leipzig, Department of Pediatric Surgery, Leipzig, Deutschland

**Background:**
Despite an awareness of the transformative potential of artificial intelligence (AI) in health care, its development in pediatric surgery seems slow. One major reason may be a lack of formal AI training. This study assesses the basic AI knowledge and the effectiveness of AI workshops (AI-WS).

**Material and methods:**
Four AI-WS were held at the International Academy of Pediatric Surgery 2024. Topics included AI principles, real-time algorithm training, and potential AI applications in pediatric surgery. Self-developed surveys consisting of eight pre-WS and nine post-WS questions were conducted, focusing on participants' AI competencies, usage, educational needs, barriers, and future perspectives.

**Results:**
Out of 57 pediatric surgeons, 53 completed both surveys. None had formal AI training. Although 90% were familiar with AI in diagnostic imaging, most had only basic knowledge of AI technology. After the workshop, participants reported a significant increase in the general understanding of AI/machine learning (ML) ($p < 0.001$). 96% stated that they were better informedabout AI/ML applications for clinical practice; 83% expressed interest in further AI training; 91% believed that AI will be more integrated into clinical practice; and over 80% anticipated that AI will improve patient outcomes.

**Conclusion:**
The AI-WS effectively enhanced pediatric surgeons' AI knowledge and their readiness to adopt AI technologies. Even though our study is limited by the relatively low sample size and a potential selection bias, our results still highlight the importance of targeted education in preparing health care professionals for AI integration. The long-term sustainability of knowledge gains, however, has to be examined in further studies.

DE GRUYTER BRILL

**Neonatal Appendicitis in Preterms: Diagnostic Challenges and Insights from a Case Series Analysis**
ID: E-DCK-435

**Kinder- und Jugendchirurgie / Paediatric and Adolescent Surgery**
**Kinderchirurgische Forschung / Paediatric Surgery Research**

Deirdre Vincent[1], Jasmin Knopf[1], Kim Heuer[2], Nina Dietze[1], Florian Kipfmüller[3], Laura Blum[1], Michael Boettcher[1]

[1]Universitätsmedizin Mannheim, Klinik für Kinder- und Jugendchirurgie, Mannheim, Deutschland, [2]Altona Kinderkrankenhaus, Pädiatrie, Hamburg, Deutschland, [3]Universitätsmedizin Mannheim, Klinik für Neonatologie und pädiatrische Intensivmedizin, Mannheim, Deutschland

**Background:**
Neonatal appendicitis represents an exceptionally rare but life-threatening surgical emergency, particularly in preterm infants. Its incidence is estimated at less than 0.1% of neonatal abdominal pathologies. Due to the nonspecific and often overlapping symptoms with necrotizing enterocolitis (NEC), diagnosis is frequently delayed until bowel perforation has occurred, resulting in peritonitis, sepsis, and high mortality. Improved recognition of characteristic clinical, laboratory, and imaging findings may allow earlier intervention and improved outcomes in this highly vulnerable population.

**Material and methods:**
A systematic retrospective case series analysis was performed using PubMed to identify all reported cases between 1980 and 2024 with the MeSH terms neonatal appendicitis, preterm infants, diagnosis, and outcomes. Data regarding gestational age, clinical presentation, laboratory parameters, imaging findings, surgical details, and postoperative outcomes were extracted and descriptively analyzed. Patterns in diagnostic delay, risk factors, and disease progression were evaluated to identify potential early indicators.

**Results:**
Twenty-one preterm neonates with histologically confirmed appendicitis were identified. The mean gestational age was 32 weeks, and the median age at diagnosis was 11 days. The most frequent presenting symptoms were progressive abdominal distension and tenderness, followed by vomiting. Laboratory abnormalities, such as leukocytosis or elevated CRP, were inconsistently observed. Radiological imaging uniformly demonstrated intraabdominal free air, indicative of intestinal perforation. At surgical exploration, all patients exhibited appendiceal perforation with secondary peritonitis.

**Conclusion:**
Neonatal appendicitis in preterm infants remains a profound diagnostic challenge, frequently mimicking NEC both clinically and radiographically. The consistently late presentation highlights the need for heightened vigilance in neonates presenting with abdominal distension and sepsis unresponsive to conventional NEC management. Early recognition of subtle clinical and laboratory patterns, coupled with low diagnostic thresholds for surgical consultation, may facilitate earlier diagnosis. Prompt surgical exploration remains the decisive factor for improving survival in this fragile population, and further multicenter analyses are warranted to refine diagnostic criteria and management algorithms.

DE GRUYTER BRILL

### COST ANALYSIS OF FOUR SURGICAL TECHNIQUES FOR PEDIATRIC INGUINAL HERNIA REPAIR IN A DUTCH HOSPITAL

ID: E-DCK-437

**Kinder- und Jugendchirurgie / Paediatric and Adolescent Surgery**
**Kinderchirurgische Forschung / Paediatric Surgery Research**

Roxanne Eurlings[1 2], Merel Kimman[2 3], Lloyd Brandts[2 3], Ruben Visschers[1 2 4], Wim van Gemert[1 2 4]

[1]Department of Pediatric Surgery, MosaKids Children's Hospital/ Maastricht University Medical Center+ (MUMC+), Maastricht, Niederlande, [2]Research Institute for Nutrition and Translational Research in Metabolism NUTRIM, Faculty of Health Medicine and Life Sciences FHML, Maastricht University, Maastricht, Niederlande, [3]Department of Clinical Epidemiology and Medical Technology Assessment (KEMTA), Maastricht University Medical Centre+ (MUMC+), Maastricht, Niederlande, [4]European Consortium of Pediatric Surgery (MUMC+, Uniklinik Aachen, Centre Hospitalier Chrétien Liège), Maastricht, Niederlande

### Background:

Pediatric inguinal hernia repair (IHR) can be performed using open repair, laparoscopic approaches such as percutaneous internal ring suture (PIRS) or conventional laparoscopic IHR (LIHR) with N-/Z-suture, and robot-assisted IHR (RIHR). While extensive literature on clinical outcomes exists, cost data remain scarce. This study aims to determine the real costs of these four techniques.

### Material and methods:

A retrospective cohort of consecutive patients with pediatric IHR in the past five years (2019–2023) was analyzed. Patients were grouped by technique. Individual one-year costs were collected, including pre-operative, per-operative, postoperative and complication costs. Kruskal-Wallis test was performed to compare costs between techniques. Multivariable-adjusted linear regression analyses were performed to identify factors associated with (non-OR) costs.

### Results:

184 patients were included in four groups: PIRS (n= 56), LIHR (n=56), Open (n=50), RIHR (n=22). Total OR cost for PIRS were significantly lower than for the other laparoscopic techniques (€1915 vs. €2414 for LIHR; p=0.001 and €3340 RIHR; p=0.000), but not for the open technique (€1915 vs. €1971; p=1.000). They were the highest for RIHR, probably due to prolonged anesthesia time, which is explained by docking of the robotic system. Other costs of the treatment journey (excluding OR costs) were significantly associated with gestational age (4.2% increase per week the patient was born earlier; p=0.001), age at surgery (7% increase per year the patient was younger; p=0.001), reducible incarceration (40.7% increase; p=0.037) and recurrence (124.5% increase; p=0.001). Hospitalization costs were significantly higher for the open technique (38.8% increase; p=0.030).

### Conclusion:

This retrospective analysis shows that the PIRS technique is related to lower OR costs. Other (non-OR) costs are highly dependent on patient characteristics and clinical outcomes. Prospective randomized studies are needed to confirm these findings and guide optimal decision making concerning surgical technique with an adequate cost-effectiveness study.

DE GRUYTER BRILL

**PIHRL-trial: Comparing PIRS with intra-corporeal purse-string suture for inguinal hernia in children**
ID: E-DCK-441

**Kinder- und Jugendchirurgie / Paediatric and Adolescent Surgery**
**Kinderchirurgische Forschung / Paediatric Surgery Research**

Roxanne Eurlings[1 2], Lore de Vreeze[3], Hamit Cakir[1 4], Stefi Nordkamp[1], Marc Dirix[1 4], Olivier Theeuws[1 4], Sylwester Gerus[5], Mateusz Palczewski[5], Joep Derikx[3], Dariusz Patkowski[5], Wim van Gemert[1 2 4], Ruben Visschers[1 2 4]

[1]Department of Pediatric Surgery, MosaKids Children’s Hospital/ Maastricht University Medical Center+ (MUMC+), Maastricht, Niederlande, [2]Research Institute for Nutrition and Translational Research in Metabolism NUTRIM, Faculty of Health Medicine and Life Sciences FHML, Maastricht University, Maastricht, Niederlande, [3]Department of Pediatric Surgery, Emma Children's Hospital, Amsterdam University Medical Center (AMC), Amsterdam, Niederlande, [4]European Consortium of Pediatric Surgery (MUMC+, Uniklinik Aachen, Centre Hospitalier Chrétien Liège), Maastricht, Niederlande, [5]Department of Pediatric Surgery and Urology, Wroclaw Medical University, Wroclaw, Polen

**Background:**
Techniques for inguinal hernia repair (IHR) in children are continuously being improved. Open repair is increasingly giving way to laparoscopy, with two possible approaches for laparoscopy: extra-peritoneally (Percutaneous Internal Ring Suturing, PIRS) or intra-peritoneally (conventional laparoscopy with purse-string suture). The aim of this trial is to compare outcomes,  e.g. recurrence and anesthesia times, of PIRS with intra-corporeal laparoscopy using a purse-string suture (LIHR).

**Material and methods:**
Subjects were prospectively included in the ‘Pediatric Inguinal Hernia Repair with Laparoscopy’ (PIHRL)-trial, to undergo surgery with PIRS or LIHR. Follow-up was conducted one year after surgery. Data concerning post-operative complications and anesthesia times were collected. Parents were asked to complete two questionnaires (POSAS and TAPQoL/TACQoL) to assess cosmetic results and quality of life.

**Results:**
177 subjects were included (PIRS n=126, LIHR n=51). There was a baseline difference in age between the groups from the different centers. Total anesthesia time was significantly shorter with PIRS (62.1±24.6min vs. 84.7±21.7min, p=0.001). One re-admittance within 30 days occurred in the LIHR group due to hemorrhage. This resolved spontaneously without the need for intervention. There was a significant difference in recurrence rate (PIRS n=2, LIHR n=4, p=0.038). One patient in the PIRS group suffered from testicular atrophy (p=0.523) and five re-interventions were necessary after PIRS due to wound complications (p=0.984).

**Conclusion:**

PIRS is safe and effective for pediatric IHR, with shorter anesthesia times and a lower recurrence rate compared to LIHR at one year follow-up. Longer follow-up and a larger sample size are necessary to confirm these results.

DE GRUYTER BRILL

**Transcriptomic profiling reveals immune-neuronal crosstalk in neurogenesis of neonatal gut**
ID: E-DCK-447

**Kinder- und Jugendchirurgie / Paediatric and Adolescent Surgery**
**Kinderchirurgische Forschung / Paediatric Surgery Research**

Sonja Diez[1], Inga Hensel[2], Luis Goncalves[2], Bruno Ghirotto Nunes[3], Manuel Besendörfer[1], Claudia Günther[2], Beate Winner[3]

[1]Universitätsklinikum Erlangen, Kinderchirurgie, Erlangen, Deutschland, [2]Universitätsklinikum Erlangen, Medizinische Klinik 1 - Gastrointestinale Pathophysiologie, Erlangen, Deutschland, [3]Universitätsklinikum Erlangen, Stammzellbiologie, Erlangen, Deutschland

**Background:**
Gastrointestinal (GI) motility disorders represent a major medical challenge, with limited therapeutic options and a high disease burden. The enteric nervous system (ENS) — a complex network of neurons and glial cells within the brain-gut-axis — plays a central role in regulating intestinal function. However, knowledge of the pediatric ENS and its developmental regulation remains limited. We hypothesize that the neonatal intestinal ENS and immune cell compartment differs from the adult one reflecting on-going gut maturation.

**Material and methods:**
Full resectates were isolated from healthy human neonatal ileum and adult ileum. Bulk RNA sequencing was performed to compare transcriptomic profiles between groups. In parallel, ENS progenitors were generated from human induced pluripotent stem cells (iPSCs) and characterized by immunostaining and calcium imaging to assess neuronal identity and functionality. Assessment of this correlation will be conducted in tissue of Hirschsprung's disease patients (results are receivable).

**Results:**
RNA sequencing of six neonatal and four adult samples revealed upregulation of genes related to innate immune signaling in neonates. Grouped analyses and validation experiments demonstrated increased IL-13 production by innate lymphoid cells (ILCs) in neonatal gut, promoting enteric neuronal differentiation via activation of the RNA-binding protein ELAVL4 (HuC/D). Immunofluorescence confirmed a higher density of HuC/D-positive neurons in neonatal compared with adult tissue, alongside distinct glial distributions. In hPSC-derived ENS cultures, IL-13 stimulation directly upregulated neurogenesis-associated gene expression and enhanced neuronal network activity.

**Conclusion:**
Our findings identify IL-13–driven immune-neuronal crosstalk as a key regulator of ENS neurogenesis in the developing human gut. We show the important role of IL-13 in gut maturation and its implication as modulator of neuronal differentiation via ELAVL4 signaling. These insights advance our understanding of pediatric ENS maturation as well as so far understudied immune-ENS communication and may uncover new therapeutic targets for congenital neuronal disorders such as Hirschsprung's disease.

DE GRUYTER BRILL

**Revised outcome prediction for neuroblastoma using deep-learning and gene-expression data**
ID: E-DCK-51

**Kinder- und Jugendchirurgie / Paediatric and Adolescent Surgery**
**Kinderchirurgische Forschung / Paediatric Surgery Research**

Moritz Markel[1], Bora Uyar[2], Vedran Franke[2], Marc Reismann[1], Angelika Eggert[3], Hedwig Deubzer[4], Steven Warmann[1], Altuna Akalin[2], Steffen Fuchs[4]

[1]Charité-Universitätsmedizin Berlin, Department of Pediatric Surgery, Berlin, Deutschland, [2]Max Delbrück Center for Molecular Medicine in the Helmholtz Association (MDC), Berlin, Deutschland, [3]University Hospital Essen, Essen, Deutschland, [4]Charité-Universitätsmedizin Berlin, Department of Pediatric Oncology and Hematology, Berlin, Deutschland

**Background:**
Neuroblastoma is a solid pediatric tumor that, despite therapeutic advances, continues to pose a clinical challenge due to its high heterogeneity. While low-risk tumors require minimal treatment, high-risk neuroblastomas are treated aggressively yet still exhibit a 5-year overall survival rate of less than 40%. The aim of this study was to develop a deep learning-based RNA classifier for precise prediction of disease outcome and early detection of relapse.

**Material and methods:**
Tumor biopsies from neuroblastoma patients at the time of diagnosis were analyzed using RNA sequencing to determine gene expression profiles. Both national and international cohorts were included (total n = 963 patients). This dataset was used to train our in-house developed deep learning model, Flexynesis, to predict overall survival (n = 674 in the training set and n = 289 in the validation set). Furthermore, specific target genes were identified and compiled into an RNA classifier, which was then correlated with established clinical risk groups.

**Results:**
A gene expression-based RNA signature consisting of six target genes was developed. When combined with clinical and biological parameters (age, stage, MYCN status), it predicted overall survival in neuroblastoma patients with a concordance index (c-index) of 0.86—surpassing the accuracy of current clinical risk stratification based on pathological and radiological criteria. Notably, the classifier identified distinct patient clusters independently of MYCN amplification status and suggested potential misclassifications within current clinical stratification.

**Conclusion:**
The developed RNA classifier enables early and accurate prognosis in neuroblastoma and may prevent misclassification, thus allowing for more effective treatment strategies. Its performance was, to a significant extent, independent of MYCN amplification status. Based on the identified gene signature, we are currently developing a targeted assay for detecting

DE GRUYTER BRILL

**Push-PEG Is Associated With Lower Costs Yet Maintains Postoperative Outcomes Compared To Pull-PEG**
ID: E-DCK-65

**Kinder- und Jugendchirurgie / Paediatric and Adolescent Surgery**
**Kinderchirurgische Forschung / Paediatric Surgery Research**

Omar Mollema[1], Hanne Verberght[1], Dianne Dinjens[2], Hamit Cakir[2], Wim van Gemert[2], Ruben Visschers[2]

[1]Research Institute of Nutrition and Translational Research in Metabolism, Maastricht University, Maastricht, Niederlande, [2]Department of Pediatric Surgery, MosaKids Children's Hospital, Maastricht University Medical Center, Maastricht, Niederlande

**Background:**
One-step percutaneous endoscopic gastrostomy (push-PEG) is safe and feasible compared to standard percutaneous endoscopic gastrostomy (pull-PEG) in pediatric patients. The literature suggests that push-PEG also reduces hospital costs as it avoids a second procedure requiring hospitalization. However, the financial consequences of perioperative and postoperative outcomes are not taken into account. Therefore, the current study aims to test the hypothesis that push-PEG is associated with lower PEG-related costs compared to pull-PEG. Furthermore, perioperative and types of PEG-related outcomes were compared.

**Material and methods:**
This retrospective, single-center cohort study was conducted at the Maastricht University Medical Center. Patients (0 – 18 years) who underwent push-PEG or pull-PEG between January 2015 and November 2024 were assessed for eligibility. Clinical and financial data were extracted from electronic medical records between December 2024 and March 2025. Data analysis was performed using the Chi-Square, Fisher's exact, or Mann-Whitney U test when appropriate. Sensitivity analyses using generalized linear models and a binary logistic regression were conducted. Statistical significance was set at $p < 0.05$.

**Results:**
Statistically significant differences were found between push-PEG (N=129) and pull-PEG (N=50). Push-PEG reduced PEG-related costs by 35% (Exp(B)=0.650, 95% CI 0.548 – 0.770, p < 0.001). No statistically significant differences were found in total postoperative complications (Exp(B)=1.262, 95% 0.774 – 2.057, p=0.350) and postoperative complications within 90 days (Exp(B)=0.629, 95% CI 0.282 – 1.406, p=0.259). Furthermore, push-PEG reduced operative time, anesthesia time, and length of hospital stay by 34%, 41%, and 50%, respectively.

**Conclusion:**
This retrospective cohort study shows that push-PEG is associated with statistically significantly lower PEG-related costs while maintaining postoperative outcomes. Furthermore, push-PEG reduces operative time, anesthesia time, and length of hospital stay for pediatric patients compared to pull-PEG. These findings support the assumption that push-PEG is more cost-effective than pull-PEG and encourage the switch to the increasingly used push-PEG as the standard PEG procedure in pediatric patients.

DE GRUYTER BRILL

### HOW TO DEAL WITH A HYPOPLASTIC URETHRA AFTER VAS?

ID: E-DCK-167

**Kinder- und Jugendchirurgie / Paediatric and Adolescent Surgery**
**Urologie - Aktuelle Strategien des refluxiven / obstruktiven Ureters / Urology – Latest Approaches to Vesicoureteral Reflux and Ureteral Obstruction**

Luisa Weil[1], Raimund Stein[1], Marietta Jank[1], Nina Younsi[1]

[1]Universitätsmedizin Heidelberg Mannheim, Kinder-, Jugend- und rebkonstruktive Urologie, Mannheim, Deutschland

**Background:**
As pediatric urologists, we are encountering an increasing number of patients receiving vesico-amniotic shunts (VAS). Consequently, we are seeing more boys with an impassable, hypoplastic urethra. Herein, we report our management of these patients in a tertiary center.

**Material and methods:**
Between October 2015 and December 2024, we reviewed our cLUTO patients. Out of 122 patients (65 with VAS), 24 had a hypoplastic urethra, where we were unable to confirm a urethral valve via cystoscopy.

**Results:**
We included 24 male patients (median follow-up of 5 years, ranging from 1 month to 9 years) with prenatally suspected lower urinary tract obstruction who received a VAS between 12 and 21 weeks of gestation (median 14 weeks). Only those with a diagnosed hypoplastic or atretic urethra via VCUG, retrograde or cystoscopy were included in the follow-up.

In 11 out of 24 cases, urethral reconstruction was performed using foreskin (plus buccal mucosa in one case). Six patients are voiding through the reconstructed urethra, while five could not void due to a neurogenic bladder (VACTERL in two cases, high ARM in two, unknown syndrome in one case). Eight patients still have their vesicostomy due to young age, four have a Mitrofanoff stoma and one patient died.

**Conclusion:**
Following VAS, we are increasingly seeing patients with a hypoplastic urethra. Urethral reconstruction has been successfully performed in those without neurogenic bladder dysfunction. However, the timing of urethral reconstruction and the correct indication remain challenging. Currently, there is no comparable data in the literature concerning urethral function after early VAS.

DE GRUYTER BRILL

**Endoscopic balloon dilatation for primary obstructive megaureter in children**
ID: E-DCK-279

**Kinder- und Jugendchirurgie / Paediatric and Adolescent Surgery**
**Urologie - Aktuelle Strategien des refluxiven / obstruktiven Ureters / Urology – Latest Approaches to Vesicoureteral Reflux and Ureteral Obstruction**

Safiullah Najem[1], Ursula Tonnhofer[1], Martin Metzelder[1], Alexander Springer[1]

[1]Medizinische Universität Wien, Wien, Österreich

**Background:**
In recent years, endoscopic high-pressure balloon dilatation (HPBD) has emerged as a minimally invasive alternative for the treatment of primary obstructive megaureter (POM). A recent meta-analysis reports a success rate of 71%. However, the optimal primary treatment of POM remains unclear, particularly in young children. This study aimed to evaluate the efficacy of HPBD in children younger than six years.

**Material and methods:**
In this retrospective study, all children aged 0 to 6 years who underwent HPBD for POM at a Northern European pediatric center over a seven-year period were analyzed. The HPBD procedure was performed using an 8.0 mm balloon (Cook Advance© 35LP PTA) over a guidewire under fluoroscopic guidance. Successful dilation was defined as follows: no further urinary infections and/or decreasing ureteric and renal dilation.

**Results:**
Between 2018 and 2025, 40 POM cases were treated, of which 29 (72.5%) underwent HPBD. The mean patient age was 1.6 years (range 0.2–5.8 years; 25 boys, 4 girls), with 22 (75.9%) under 24 months. The mean preoperative ureteral diameter was 16.5 mm (range 11–24 mm). HPBD was performed on the left side in 17 (58.6%) patients, on the right side in 12 (41.4%), and bilaterally in 1 patient. Only six (20.7%) patients received a JJ stent. No intraoperative complications occurred. One patient (3.4%) required ureteral reimplantation, and one (3.4%) required a second HPBD procedure. The median follow-up was 33.5 months (range 2–78.6 months).

**Conclusion:**
Endoscopic HPBD using an 8.0 mm balloon without JJ stenting is a safe, feasible and durable. It is a promising minimal invasive primary treatment option for POM in children under six years of age.

DE GRUYTER BRILL

**High Impact, Low Cost: A Practical Model for Urological Education and Practice**
ID: E-DCK-379

**Kinder- und Jugendchirurgie / Paediatric and Adolescent Surgery**
**Urologie - Aktuelle Strategien des refluxiven / obstruktiven Ureters / Urology – Latest Approaches to Vesicoureteral Reflux and Ureteral Obstruction**

Andrea Schmedding[1]

[1]Städtisches Klinikum Braunschweig, Kinderchirurgie und Kinderurologie, Braunschweig, Deutschland

**Background:**
Pyeloplasty and antireflux surgery are standard procedures in pediatric urology. According to the National Competence-Based Learning Objectives Catalogue for Medicine (NKLM), the principles of these surgical techniques must be taught as part of medical education. Surgical procedures are particularly well suited for simulation using models that allow hands-on practice of techniques. However, many hospitals and surgeons face budgetary constraints that limit their ability to acquire commercial training models. This study aims to develop a suitable model that can be produced at low cost and with minimal technical effort. Furthermore, it presents the model's first use in undergraduate medical education and its evaluation by the students.

**Material and methods:**
A literature review was performed to identify feasible low-cost models representing the anatomical structures of the kidney, renal pelvis, and ureter. Among the various options found, a simple model constructed using two balloons was selected due to its simplicity, accessibility, and cost-effectiveness. This prototype was subsequently refined and adapted for use in undergraduate medical education. A second model for the bladder and the ureter was derived from the first model. The model was implemented in small-group teaching sessions to facilitate hands-on learning of urological anatomy and basic surgical principles. Following its use in teaching, a structured evaluation was conducted to assess the model's educational effectiveness and student acceptance.

**Results:**
For pyeloplasty training, a model was developed using kidneys made of industrial modeling clay, which can be easily shaped at 60 °C and retains its form at room temperature. The renal pelvis and ureter were represented by two balloons. The balloon representing the renal pelvis was attached using a simple bag clip, allowing for easy adjustment. The total cost of the model was approximately €2.50. Shaping each kidney took about five minutes, with an additional 5–10 minutes required for balloon assembly.
For ureteral reimplantation and antireflux surgery, a second model was constructed using four balloons. One balloon simulated the detrusor muscle, while a second balloon inside it represented the bladder urothelium. Elongated balloons were used to simulate the ureters and were anchored within the inner (urothelium) balloon.
In the teaching sessions, medical students first performed an antireflux procedure, followed by ureteral reimplantation, and finally a pyeloplasty. All procedures were carried out as open surgeries on the models. The total time required to complete all three procedures was approximately 2.5 hours. A

subsequent evaluation indicated that the models were highly suitable for teaching fundamental surgical principles.

**Conclusion:**
Evaluated low-cost models in surgery provide effective simulations of the pyeloplasty, antireflux procedure and ureteral reimplantation. Using a laparoscopic simulator, existing exercises can be enhanced with these affordable models to practice surgical skills. Even departments with limited technical resources can create these models, providing valuable training opportunities for students and staff.

DE GRUYTER BRILL

**Vesicoureteral Reflux and Surgical Techniques at the Ureteral Orifice: Standard or Spectrum?**
ID: E-DCK-401

**Kinder- und Jugendchirurgie / Paediatric and Adolescent Surgery**
**Urologie - Aktuelle Strategien des refluxiven / obstruktiven Ureters / Urology – Latest Approaches to Vesicoureteral Reflux and Ureteral Obstruction**

Andrea Schmedding[1], Sabine Drossard[2]

[1]Städtisches Klinikum Braunschweig, Kinderchirurgie und Kinderurologie, Braunschweig, Deutschland,
[2]Universitätsklinik Würzburg, Chirurgie I: Abteilung für Kinderchirurgie, Würzburg, Deutschland

**Background:**
Vesicoureteral reflux (VUR) is one of the most common congenital urological anomalies in childhood. In the general population, symptomatic VUR is estimated to occur in approximately 1–2% of children. However, among children presenting with febrile urinary tract infections, VUR can be detected in up to 30–40% of cases. Despite advances in diagnostic methods and treatment options, there is still no consensus on the optimal management strategy, with surgical, endoscopic, and conservative approaches all being employed. In recent years, minimally invasive techniques have gained increasing importance, and robotic-assisted surgery is now also in use. This study provides an overview of the management for children and adolescents with VUR and surgical management of the ureteral orifice in Germany, based on an analysis of billing data from the past six years.

**Material and methods:**
We conducted a retrospective analysis of data provided by the InEK (Institute for the Hospital Remuneration System) for the years 2019 to 2024. These datasets reflect the cumulative annual statistics for patients insured under the German statutory health insurance (GKV). For the years 2021 to 2024, departmental codes were also taken into account.
All inpatient cases under the age of 18 with a primary diagnosis of vesicoureteral reflux (ICD codes N11.0, N13.7, Q62.7) were included. Each annual dataset generated contained information on age distribution, average length of hospital stay, and lists of primary diagnoses (ICD), secondary diagnoses (ICD), procedures (OPS), and DRGs associated with the cohort.
In a second analysis, the cohort was further defined by the presence of the following procedure codes: OPS 5-568.9 (isolated antireflux surgery, e.g., according to Lich-Gregoir), 5-569.6 (injection for ureteral orifice insufficiency), and 5-568.d, 5-568.8, 5-568.e (ureteral reimplantation).

**Results:**
Between 2019 and 2024, an average of 2,517 inpatient cases per year were treated in Germany with a primary diagnosis of vesicoureteral reflux. Of these, 60% involved female patients. In terms of age distribution, 15.3% of the children were younger than one year, 47.4% were 1–2 years old, 12.71% were 3–5 years, 8.77% were 6–9 years, 10.38% were 10–14 years, and 5.45% were 16–17 years old. Patients treated in pediatric surgery departments were younger on average than those treated in urology departments.

The average length of hospital stay was 2.8 days overall, 3.0 days in pediatric surgery, and 3.6 days in urology.
In 10.7% of cases, a duplicated kidney (duplex system) was documented, renal agenesis in 0.7%, and renal insufficiency in 0.8%. Anorectal malformation was listed as a secondary diagnosis in 0.2% of cases. Among these patients, 23% underwent subureteric injection, 10% received ureteral reimplantation, and 13% underwent antireflux surgery.
In 2021, 42% of cases were billed through pediatric surgery departments; this proportion increased to 48% by 2024. At the same time, the proportion treated in urology departments decreased from 10% to 7%, while other specialties decreased from 48% to 45%.
To allow for a more detailed analysis of the surgical techniques, the procedures were re-evaluated independently of the primary diagnosis.
On average, 693 subureteric injections were performed annually (total: 4,157). Case numbers fluctuated year by year without a clear trend. Girls accounted for 70% of cases. 9.1% of procedures were performed in the first year of life, and 40.1% in the second and third years. 73% of these cases were billed under pediatric surgery, and 8% under urology.
Regarding antireflux surgery according to Lich-Grégoir, an average of 403 procedures were performed annually (total: 2,419), of which 9.8% were laparoscopic, 33.2% were classified as "other," and 57.2% were open procedures. The use of robotic-assisted surgery was first documented in 2022, with 9 cases, increasing to 22 cases in 2024. The proportion of laparoscopic procedures rose significantly over the years, reaching 19.7% in 2024.
An average of 496 ureterocystoneostomies (UCN) were performed per year (total: 2,976), with 17.2% performed laparoscopically. Robotic-assisted surgery was introduced in 2022, and a total of 1% of these procedures were robotically assisted.

**Conclusion:**
The analysis highlights that vesicoureteral reflux remains a frequently treated condition in childhood in Germany. While minimally invasive therapy via transurethral subureteric injection predominates numerically, the proportion of laparoscopic procedures for reconstructive surgeries is steadily increasing, with initial cases now being performed using robotic assistance. The majority of treatments are performed within pediatric surgery departments. Overall, the results indicate a growing adoption of minimally invasive techniques and a shift in care provision toward pediatric surgery.

DE GRUYTER BRILL

**Where is the gold standard? - Pyeloplasty in Children**
ID: E-DCK-374

**Kinder- und Jugendchirurgie / Paediatric and Adolescent Surgery**
**Urologie - Chirurgie von Nierenerkrankungen: konventionell? Laparoskopisch? Robotisch? / Urology – Kidney Surgery: Conventional? Laparoscopic? Robotic?**

Andrea Schmedding[1], Fritz Kahl[2], Elisabeth Ammer[2], Ludger Tüshaus[3]

[1]Städtisches Klinikum Braunschweig, Kinderchirurgie und Kinderurologie, Braunschweig, Deutschland, [2]Universitätsmedizin Göttingen, Klinik für Allgemein-, Viszeral- und Kinderchirurgie, Göttingen, Deutschland, [3]Universitätsklinikum Schleswig-Holstein, Kinderchirurgie, Lübeck, Deutschland

**Background:**
The gold standard for pyeloplasty in children is defined as minimally invasive surgery (MIS), either traditional or robotic-assisted (RA). This study aims to determine the extent to which this gold standard has prevailed in Germany.

**Material and methods:**
Methods: The InEK publishes annual and interim cumulative statistics on procedures performed in German hospitals. Data were collected for the years 2019 to 2024, including all cases involving procedure 5-557.4 (pyeloplasty).

**Results:**
From 2019 to 2024, a total of 4,428 pyeloplasties were performed, with 29.2% occurring in the first year of life. In 2019, 57.1% of procedures were open surgical (OS), decreasing to 47.1% by 2024. The use of surgical robots increased from 7.2% in 2019 to 18% in 2024, with 75.1% of robotic procedures performed on patients aged 10-17 years. Retrograde urography was conducted in 9.8% to 12.8% of cases. The percentage of children receiving ureteral stents rose from 50.3% in 2019 to 57.7% in 2024. Between 1-1.7% of children received transfusions from 2019 to 2023. The average length of stay (LOS) for all operated children decreased from 8.3 days in 2019 to 6.6 days in 2024, with average LOS of 7.4 days with OS, 5.8 days with MIS, and 5.3 days with RA surgery in 2024.

**Conclusion:**
Although laparoscopic pyeloplasty is seen as gold standard in literature, it has not yet become the most common surgical technique in childhood in Germany. Robotic pyeloplasty has been gaining importance in recent years.

**The first 20 esophageal atresia patients treated by magnetic compression anastomosis using ConnectEA**
ID: E-DCK-110

**Kinder- und Jugendchirurgie / Paediatric and Adolescent Surgery**
**Varia / Miscellaneous**

Oliver Muensterer[1], Anne-Sophie Holler[1], Dan Laser[2], Michael Harrison[3]

[1]Dr. von Haunersches Kinderspital, LMU Klinikum, Kinderchirurgie, München, Deutschland, [2]myka labs, San Francisco, Vereinigte Staaten, [3]UCSF, Pediatric Surgery, San Francisco, Vereinigte Staaten

**Background:**
Management of long-gap and other forms of complex esophageal atresia (EA) remains a challenge. Magnetic compression anastomosis (MCA) is an endoscopic procedure that has the potential of limiting the need for more invasive surgery. We developed specially-shaped high-power magnets (connect EA™) that have been shown to facilitate a secure, reliable esophageal anastomosis in preclinical studies. The connect EA™ MCA system was employed in a large spectrum of patients with complicated EA under compassionate care ethics. This report details our experience, including challenges, pitfalls, and outcomes.

**Material and methods:**
A retrospective review of all patients in whom the connect EA™ System was employed with the intention of creation of an esophageal MCA in EA patients at our institutions was performed. Patient demographics, success in facilitating a patent MCA, intraoperative and postoperative parameters, subsequent interventions including dilatations, complications and outcomes were recorded and evaluated.

**Results:**
From 2019 through 2025, a total of 20 EA patients were identified who had endoscopic intraluminal placement of the connect EA™ magnets (see table below). All patients had severe co-morbidities that placed them at higher risk for surgical repair and prolonged general anesthesia. In this cohort, 16 interventions were successful and led to an esophageal anastomosis within 3 to 14 days after the procedure. In 4 cases, the magnets did not mate or separated in the first 24 hours after the procedure. These patients underwent conventional thoracoscopic (n=2) or open surgical (n=2) anastomosis. One MCA patient developed a recurrent tracheoesophageal fistula. Patients had between 3 and 12 dilatations over a follow-up period of up to 6 years. None of the patients required stent placement or resection/anastomosis for stricture. Two patients developed leaks. All patients eventually established full oral nutrition.

**Conclusion:**
This report demonstrates that MCA using the connect EA™ system is feasible and safe with favorable outcomes in complex EA patients with severe co-morbidities. The complication profile is similar to the conventional technique. In patients who may not tolerate long operative and anesthesia times, the device offers considerable benefit over conventional handsewn esophageal anastomosis. Future prospective and comparative studies should be performed to substantiate these findings.

| Patient | Sex (m/f) | EA type | GA birth (weeks) | Birth weight (grams) | Approximation (y/n), approach | MCA age (days) | Time to patency (postop days) | Closure gastrostomy (days) age | Dilatations (n) | Follow-up (years) |
|---|---|---|---|---|---|---|---|---|---|---|
| **1** | f | A | 35 | 1830 | y thoracoscopic | 51 | 7 | 122 | 4 | 6 |
| **2** | m | B | 33 | 1980 | y thoracoscopic | 58 | 12 | 297 | 4 | 6 |
| **3** | f | C | 42 | 3700 | y thoracoscopic | 112 | 14 | 273 | 5 | 5 |
| **4** | f | A | 36 | 1965 | y thoracoscopic | 76 | 6 | 132 | 3 | 4 |
| **5** | m | B | 38 | 3210 | y thoracoscopic | 175 | Unsuccessful | 385 | >15 | 4 |
| **6** | m | C | 36 | 2000 | y open | 138 | Unsuccessful | 270 | 3 | 4 |
| **7** | m | C | 40 | 3190 | y open | 27 | 14 | 124 | 5 | 4 |
| **8** | f | C | 34 | 1670 | y open | 203 | 8 | 455 | 11 | 3 |
| **9** | m | A | 38 | 2690 | n | 15 | 6 | 118 | 5 | 3 |
| **10** | m | A | 30 | 1600 | y laparoscopic | 156 | 4 | 540 | 7 | 3 |
| **11** | f | A | 37 | 1900 | y thoracoscopic | 34 | 7 | 320 | 12 | 3 |
| **12** | m | C | 26 | 850 | y thoracoscopic | 82 | 9 | 210 | 4 | 2 |
| **13** | f | B | 27 | 825 | n | 143 | 14 | 284 | 9 | 2 |
| **14** | f | C | 35 | 2020 | y open | 129 | 14 | not closed | 9 | 2 |
| **15** | f | C | 37 | 2530 | y open | 14 | Unsuccessful | not closed | 9 | 2 |
| **16** | m | A | 38 | 2920 | y thoracoscopic | 39 | 9 | 125 | 9 | <1 |
| **17** | m | C | 36 | 2600 | y open | 36 | 14 | not closed | 7 | <1 |
| **18** | f | A | 38 | 2840 | n | 32 | Unsuccessful | not closed | 3 | <1 |
| **19** | f | C | 32 | 1360 | y open | 143 | 10 | not closed | 3 | <1 |
| **20** | m | A | 38 | 2645 | y thoracoscopic | 102 | | not closed | | <1 |
| | **10 m<br>10 f** | **8 A<br>3 B<br>9 C** | **median 36 [26-42]** | **median 2010 [825-3210]** | **15 y<br>5 n<br>9 thoracoscopic** | **median 82 [14-175]** | **median 9.5 [4-14]** | **median 273 [125-540]** | **median 5 [3->15]** | **median 3 [<1-6]** |

DE GRUYTER BRILL

**Y-V MEATOPLASY; A SIMPLE NOVEL TECHNIQUE TO CORRECT MEATAL STENOSIS**
ID: E-DCK-15

**Kinder- und Jugendchirurgie / Paediatric and Adolescent Surgery**
**Varia / Miscellaneous**

Michael Sennert[1], Mohammed Fawzy[1], Hanno Wirmer[1], Carsten Graumann[1], Ahmed Hadidi[1]

[1]Sana Klinikum Offenbach, Kinderchirurgie, Offenbach am Main, Deutschland

**Background:**
Meatal stenosis is a common complication following hypospadias repair. Dilatation or meatotomy is usually not effective and may result in recurrence of symptoms. The aim of this work is to report our experience with Y-V Meatoplasty, a simple novel technique to correct post-operative meatal stenosis after hypospadias surgery with up to 8 years follow up.

**Material and methods:**
A small, inverted V skin flap is elevated with the angle at the tip of the meatus. Each limb is 3-5 mm long. A wedge of the narrow meatus, underneath the flap, is excised creating a wide meatus. The tip of the inverted V is advanced distally and sutured to the angle inside the wide new meatus. The aim is to interrupt the ring suture line by introducing the V skin flap into the ring. This minimizes the chances of further ring contraction.
The technique has been used in 57 patients with symptomatic post-operative meatal stenosis in the period from 2017 till 2024. The median age was 53 months (range 17 months to 44 years). Median Follow up was 4 years (range 3 months to 8 years).

**Results:**
52 patients recovered well without further symptoms or problems. Three children complained of mild dysuria after surgery that disappeared within a month after surgery without further intervention. Two patients (3.5%) early in the study required further surgery due to inadequate excision of the stenotic ring. There were no cases of postoperative urinary tract infections.

**Conclusion:**
This study describes the Y-V Meatoplasty, that represents a simple effective technique to successfully correct post-operative ring meatal stenosis with a low complication rate of 3.5%.

DE GRUYTER BRILL

**Physical Endurance and Psychosocial Well-Being after Resection of Congenital Lung Lesions**
ID: E-DCK-245

**Kinder- und Jugendchirurgie / Paediatric and Adolescent Surgery**
**Varia / Miscellaneous**

Sinja Lambrecht[1], Julia Elrod[1], Michaela Klinke-Petrowsky[1], Meike Weis[2], Johannes Boettcher[3], Richard Martel[1]

[1]Medizinische Fakultät Mannheim der Universität Heidelberg, Universitätsmedizin Mannheim, Klinik für Kinder- und Jugendchirurgie, Mannheim, Deutschland, [2]Medizinische Fakultät Mannheim der Universität Heidelberg, Universitätsmedizin Mannheim, Klinik für Radiologie und Nuklearmedizin, Mannheim, Deutschland, [3]Universitätsklinikum Hamburg-Eppendorf, Kinder- und Jugendpsychiatrie, Hamburg, Deutschland

**Background:**
Congenital lung lesions (CLL) are typically treated with surgical resection to prevent complications. While resection in symptomatic newborns is standard, management of asymptomatic cases remains controversial. Early intervention may support lung growth during alveogenesis and prevent infections, which increase after age two in CPAM patients.

**Material and methods:**
At University Medical Center Mannheim, all patients with CLL undergoing surgical or conservative management between 2000 and 2023 were analyzed. Operative cases included open and thoracoscopic resection. Outcomes assessed included physical endurance via 6-minute run test and psychosocial well-being using standardized questionnaires (PedsQL, SDQ, PACS, EQ-5D-3L) for children and parents.

**Results:**
194 children were included (120 CPAM, 53 pulmonary sequestration, 14 hybrid lesions, 6 congenital lung emphysema, 6 others). Surgical therapy was performed in 162 (83.5%) and conservative management in 32 (16.5%) children. Postnatal LOHS was shorter in the conservative group (22 days vs. 6 days; *p<.0001*), as was ICU-need (78.2% vs. 53.6%; *p<0.01*). rFLV was lower in surgical patients (50.8% vs. 78.9%; *p=0.015*). Open resection (n=120) was associated with higher ICU needs, complications (46.4% vs. 31.0%; *p<0.05*), and LOHS (29.0 vs. 4.0 days; *p<.0001*) than thoracoscopic (n=31). After propensity score matching the significant difference in ICU-need such as rFLV was no longer detectable. No significant differences were found in physical endurance or psychosocial well-being in both parents and children

**Conclusion:**
Long-term outcomes showed equivalent physical endurance and psychosocial well-being after resection of congenital lung lesions compared with conservative management. These results support surgery as a preventive measure against malignancy and infection without compromising fitness or psychosocial well-being.

DE GRUYTER BRILL

**Robot-Assisted Surgery in Neonates: Opportunities and Limitations Demonstrated by Duodenal Atresia**
ID: E-DCK-262

**Kinder- und Jugendchirurgie / Paediatric and Adolescent Surgery**
**Varia / Miscellaneous**

Mareike Jassyk[1 2], Jochen Hubertus[1 2]

[1]Marien Hospital Witten, Kinderchirurgie, Witten, Deutschland, [2]Ruhr-Universität Bochum, Kinderchirurgie, Bochum, Deutschland

**Background:**
Duodenal atresia, with an incidence of approximately 1 in 7.000 live births, represents the second most common congenital intestinal anomaly. Surgical correction within the first days of life is aimed for. Minimally invasive duodeno-duodenostomy is considered one of the more complex laparoscopic procedures in neonates. Robot- assisted surgery is increasingly being integrated into pediatric surgical practice, offering enhanced precision and visualization. However, this approach also entails specific technical challenges and limitations that must be put into consideration.
**Objective**
To highlight the potential benefits and current limitations of robot- assisted surgery in neonates a case of duodenal atresia will be presented. Special emphasis is placed on the technical and anatomical considerations unique to the neonatal situs.

**Material and methods:**
**Case Report**
The patient presented is a full-term female neonate (gestational age 37 + 1 weeks) with a birth weight of 2,485 g. Prenatal imaging suggested duodenal atresia, which was confirmed postnatally. A robot-assisted laparoscopic duodeno-duodenostomy was performed on the second day of life. The patient was placed on a vacuum mattress with the lower extremities positioned downward to optimize access. The Da Vinci Si Robotic System was docked with three operative arms. The duodeno-duodenostomy was completed after 90 minutes of operative time.
The postoperative clinical course was uneventful: the first passage of stool occurred on postoperative day 3, full enteral feeding was achieved by day 9, and discharge followed on day 13.
*Fig.1 Postnatal X-ray “double bubble” sign*
*Fig.2 Trocar Placement*
*Fig.3 Duodeno-duodenostomy using the Da Vinci system*

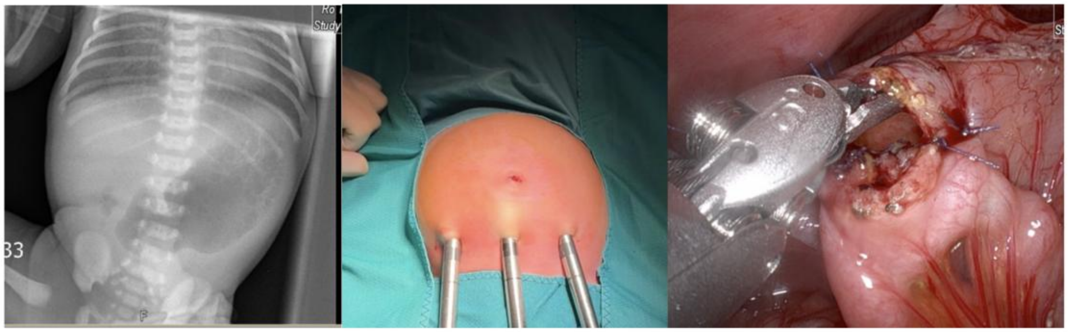

Fig.1 Fig.2 Fig.3

**Results:**
Robot- assisted surgery provides significant technical advantages in the neonatal setting, particularly through enhanced instrument articulation with seven degrees of freedom and high-definition 3D visualization. Nevertheless, the unique anatomical characteristics of neonates require meticulous planning to minimize the risk of iatrogenic injury.

**Conclusion:**
Robot- assisted surgery represents a promising advancement in minimally invasive pediatric surgery, particularly in complex neonatal procedures. The approach offers superior dexterity and visualization, potentially improving surgical outcomes.
However, its application in neonates necessitates detailed preoperative planning regarding patient positioning, trocar placement, and system docking, and depends on accurate knowledge of the patient's unique anatomical features.

DE GRUYTER BRILL

**Rectal biopsy forceps: affordable, sustainable method for diagnosing Hirschsprung Disease**
ID: E-DCK-318

**Kinder- und Jugendchirurgie / Paediatric and Adolescent Surgery**
**Varia / Miscellaneous**

Sigrid Blom[1], Manouk Backes[1], Ivo de Blaauw[1], Floris Ferenschild[1], Stefanie Märzheuser[2], Judith Lindert[2], Roxana Rassouli-Kirchmeier[1]

[1]Radboudumc Njjmegen, Department of Paediatric Surgery, Njjmegen, Niederlande, [2]University Medicine Rostock, Department of Paediatric Surgery, Rostock, Deutschland

**Background:**
Rectal suction biopsies (RSB) are commonly used in young children, allowing bedside rectal biopsy without the need for anesthesia. However, some authors report challenges with handling the suction device. The cartouche is non-reusable, and the procedure requires two trained users. Recently, supply chain issues have highlighted the need for an alternative biopsy method for patients suspected of Hirschsprung Disease.
At our center in the Netherlands, we have been using rectal biopsy forceps (RBF) since 2000. A center in Germany adopted this technique based on our experience in 2023.

**Material and methods:**
We conducted a cross-sectional review of electronic hospital records, analyzing patient demographics, biopsy adequacy, procedural challenges, and outcomes. Data were analyzed using SPSS version 26.

**Results:**
A total of 136 rectal biopsies were performed using forceps across two colorectal centers. Four complications were reported: one perforation and three instances of bleeding.

**Conclusion:**
The rectal biopsy forceps method is a simple, cost-effective, and sustainable bedside technique for obtaining rectal biopsies in infants without anesthesia. Given the low complication rate, it presents a safe and reliable alternative for diagnosing Hirschsprung Disease.

Abstracts – DCK 2026 – 143rd Congress of the German Society of Surgery – Leipzig, April 22–24 • DOI 10.1515/iss-2026-2003
Innov Surg Sci 2026; 11 (Special Suppl 1): s270–s329

DE GRUYTER BRILL

**Ventriculobiliary Shunt Implantation as a Salvation Procedure in Posthemorrhagic Hydrocephalus**
ID: E-DCK-363

**Kinder- und Jugendchirurgie / Paediatric and Adolescent Surgery**
**Varia / Miscellaneous**

Carola Hörz[1], Christian Kruppa[1], Guido Fitze[1]

[1]University Clinic Carl Gustav Carus Dresden, Dresden, Deutschland

**Background:**
This case report presents ventriculobiliary shunt implantation as an alternative for hydrocephalus treatment in a complex case where standard methods failed.

**Material and methods:**
A preterm infant (27+2 weeks, 1250 g) underwent successful surgery for a small omphalocele but later developed necrotizing enterocolitis and posthemorrhagic hydrocephalus, requiring twelve surgeries within the first eleven months of life. Wiedemann-Beckwith syndrome was confirmed during the course of the hospital stay.
Establishing cerebrospinal fluid drainage was challenging. Ventriculoperitoneal (VP) shunt placement failed due to intra-abdominal adhesions and the patient required multiple surgical revisions. Ventriculocisternostomy and ventriculoatrial (VA) shunt placement were also unsuccessful due to vascular complications.

**Results:**
Ultimately, a ventriculobiliary shunt was implanted. Until now, during a four-year follow-up, no signs of shunt insufficiency were observed.

**Conclusion:**
Ventriculobiliary shunting proved effective when VP and VA shunts were not feasible. Compared to VA shunts, it has a lower risk of severe complications while maintaining similar overall complication rates to standard methods. Case reports document long-term functionality, with one reporting up to 20 years without complications. However, data remain limited, and further studies are needed.
Given its comparable risk profile and long-term efficacy, ventriculobiliary shunting can be considered a viable alternative in special cases where conventional shunt placement fails.

DE GRUYTER BRILL

**Incidence and predictive factors of perforated appendicitis in children during COVID-19 pandemic**
ID: E-DCK-479

**Kinder- und Jugendchirurgie / Paediatric and Adolescent Surgery**
**Varia / Miscellaneous**

Niko Keller[1], Manuel Kramer[2 3], Thomas Krebs[1], Frank-Martin Häcker[1 4]

[1]Ostschweizer Kinderspital, Kinderchirurgie, St. Gallen, Schweiz, [2]HOCH Health Ostschweiz, Kantonsspital St.Gallen, Orthopädie, St. Gallen, Schweiz, [3]Ostschweizer Kinderspital, Kinderorthopädie, St. Gallen, Schweiz, [4]Universitäts-Kinderspital beider Basel, Kinderchirurgie, Basel, Schweiz

**Background:**
The COVID-19 pandemic disrupted healthcare systems globally, potentially delaying medical care for acute surgical conditions like appendicitis. It is commonly known that depending on the duration of symptoms, perforated appendicitis can cause serious perioperative (e.g., peritonitis or sepsis) and postoperative complications (e.g., intraabdominal abscess formation, mechanical obstruction and wound infection). These, in turn, possibly impair long-term quality of life. The aim of this study was to assess whether the COVID-19 pandemic and pandemic related health care restrictions had an influence on the incidence and morbidity of perforated appendicitis in Eastern Switzerland. Furthermore, predictive parameters for perforated appendicitis were investigated and it was attempted to apply them to improve management of this potentially life-threatening condition.

**Material and methods:**
A retrospective, single center cohort analysis was performed including all children and adolescents under the age of 18 who underwent appendectomy for acute appendicitis between January 1$^{st}$, 2018, and March 31$^{st}$, 2023. Patients were divided into three periods: pre- (September 2018 – March 2020), peri- (March 2020 – September 2021), and post-pandemic (September 2021 – March 2023). Data on demographics, clinical presentation, laboratory values, duration of symptoms, pathological findings, readmission on emergency room (ER) before treatment, hospital length of stay and complications were analyzed using R, version 4.2.2 (R Core Team, 2022).

**Results:**
The incidence of perforated appendicitis was not significantly different across the pre- (23.6%), peri- (22.5%) and post-pandemic (29.0%) periods ($p=0.331$). Duration of symptoms was significantly shorter during the pandemic (38.24h) compared to the non-pandemic (49.64h) periods ($p<0.05$). There was no statistic significant difference in intraoperative, postoperative and long-term complication rates during the pandemic compared to the non-pandemic periods. Predictive parameters for perforated appendicitis are CRP (Cutoff 46.5mg/l), relative neutrophile count (Cutoff 79,4%) and duration of symptoms (Cutoff 30h).

**Conclusion:**
In Eastern Switzerland, the COVID-19 pandemic did not lead to an increased incidence of perforated appendicitis, higher morbidity or more complications. On the contrary, there was a reduction of perforation rates due to significantly reduced duration of symptoms, potentially due to reduced waiting time in the ER.

DE GRUYTER BRILL

We identified predictive parameters (C-reactive protein, relative neutrophile count and duration of symptoms) for perforated appendicitis in children and adolescents. This could guide clinicians to a more effective case management, including improved planning, an optimized selection of operation techniques and reduced intra- and postoperative complication rates.

DE GRUYTER BRILL

### COMPARISON OF LAPAROSCOPIC AND OPEN SURGICAL OPTIONS FOR CONGENITAL DOUDENAL OBSTRUCTION

ID: E-DCK-508

**Kinder- und Jugendchirurgie / Paediatric and Adolescent Surgery**
**Varia / Miscellaneous**

Christian Kruppa[1], Ulugbek Hamroyec[2], Guido Fitze[3], Bakthtior Ergashew[4]

[1]uniklinikum der tu dresden, Kinder- und Jugendchirurgie, Dresden, Deutschland, [2]Republican Perinatal Center, Tashkent, Uzbekistan, [3]Uniklinikum Dresden, Dresden, Deutschland, [4]Republican Perinatal Center, Tashkent, Tashkent, Uzbekistan

**Background:**
We compare the results of laparoscopic surgery with those of open surgery. The characteristics of the various principles should be determined on the basis of the time of surgery, the clinical course, the postoperative diet, the complications and the cosmetics.

**Material and methods:**
In this retrospective study, data from 100 patients from two centers in Uzbekistan and Germany were collected. Of these, 28 laparoscopic and 39 open operations were performed as duodenoduodenostomies. In addition, for 33 patients a duodenojejunostomy was performed.

**Results:**
Patients who underwent laparoscopic surgery had a higher birth weight and a lower proportion of trisomy 21 diagnoses (3% vs. 33%). The operating times for laparoscopic and open duodenojejunostomy were almost identical at about 116 minutes. The open duodenoduodenostomy was significantly faster with an average of 71 minutes.
A total of 34 patients from the open group were operated on via a minimal transumbilical approach. This did not lead to a prolongation of the operation, regardless of the method used.
One patient from the laparoscopy group had to be operated on due to persistent stenosis. Children with transanastomotic splinting had fewer aspirations (8% compared to 32%). The time to reach full enteral nourishment was similar for all groups between 12 and 14 days.

**Conclusion:**
The different surgical approaches and methods are safe options with comparable results. Transanastomotic splinting reduces the frequency of aspiration events. Cosmetically, the transumbilical approach is a strong competitor to laparoscopy.

DE GRUYTER BRILL

**SILS vs. 3PL in Pediatric Appendectomy: Analysis of Preoperative Risk Factors and Outcomes**
ID: E-DCK-77

**Kinder- und Jugendchirurgie / Paediatric and Adolescent Surgery**
**Varia / Miscellaneous**

Britt Sievers[1 2], Maximilian Bernhardt[1 2], Andreas Meinzer[1 2], Katja Reischig[1 2], Robert Bergholz[1 2], Gennadii Ivanov[1 2]

[1]Universitätsklinikum Schleswig-Holstein, Klinik für Allgemeine Chirurgie, Viszeral-, Thorax-, Transplantations- und Kinderchirurgie, Kinderchirurgie, Kiel, Deutschland, [2]Universitätsklinikum Schleswig-Holstein, Kurt-Semm-Zentrum für laparoskopische und roboterassistierte Chirurgie, Kiel, Deutschland

**Background:**
Laparoscopic appendectomy is the standard operative treatment for acute appendicitis in children. Alongside the established three-port laparoscopy (3PL), single-incision laparoscopic surgery (SILS) has gained popularity. Current evidence indicates that both approaches are comparably safe, with minor advantages of SILS regarding cosmetic outcome and length of hospital stay. However, individual studies suggest that factors such as elevated body mass index (BMI), transmural perforation, or male sex may increase the risk of conversion, particularly in SILS. These associations have not been systematically validated in pediatric populations. This study aimed to evaluate the impact of preoperative risk factors on surgical outcomes in SILS and 3PL within an unselected pediatric cohort.

**Material and methods:**
This retrospective study included all patients under 18 years of age who underwent appendectomy between 2015 and 2025 and for whom parental consent for retrospective data analysis was obtained. Preoperative parameters assessed were: Age, BMI, CRP, sodium and suspected perforation/abscess. Endpoints included morbidity and mortality, conversion or extension of the incision, operative time, and length of hospital stay. Associations and interactions between risk factors and surgical approach were analyzed using logistic and linear regression.

**Results:**
55 patients were included: (3PL n= 26 and SILS n=29) There were no significant differences in operative time or conversion rate between the two approaches (3PL: 88.5 min [IQR 53.0 min] vs. SILS: 78.0 min [IQR 42.0 min], $p = 0.500$). However, hospital stay was significantly shorter after SILS (3PL: 7 days [IQR 2.5 days] vs. SILS: 4 days [IQR 3.25 days], $p = 0.003$). Younger children showed an overall increased risk of conversion regardless of surgical method. BMI was identified as a significant predictor of conversion in the overall regression analysis, but when patients were dichotomized at a calculated BMI threshold of 17 to test for a potential cut-off effect, this association was not confirmed. Other preoperative markers such as CRP, sodium, or sonographic suspicion of perforation showed no relevant influence on conversion rate, operative time, or hospital stay. Histological classification into uncomplicated and complicated appendicitis was likewise evaluated in logistic and linear regression models and did not emerge as a significant factor (all $p > 0.6$), indicating that the inclusion of complicated cases did not substantially confound the results.

**Conclusion:**
SILS appears similar to 3PL in pediatric appendectomy and is associated with a shorter hospital stay. While preoperative factors such as age or BMI affect the likelihood of conversion, our data does not allow reliable preselection of the surgical method. These findings demonstrate that even in an unselected cohort including complicated appendicitis, no clear exclusion criteria for SILS exist. The added value of this study lies in the interaction-based analysis of preoperative parameters in a pediatric setting, suggesting that the choice of approach should remain primarily individualized and experience- based.
